# Improving the patient-reported outcome sections of clinical trial protocols: a mixed methods evaluation of educational workshops

**DOI:** 10.1007/s11136-022-03127-w

**Published:** 2022-05-12

**Authors:** Madeleine T. King, Margaret-Ann Tait, Rachel Campbell, Fabiola Müller, Claudia Rutherford, Corinna Beckmore, Sophie Chima, Danette Langbecker, Joanne Shaw, Rebecca Mercieca-Bebber

**Affiliations:** 1 Sydney Quality of Life Office, School of Psychology, University of Sydney, Sydney, NSW 2006 Australia; 2 Department of Medical Psychology, Amsterdam University Medical Centers, University of Amsterdam, Amsterdam Public Health Research Institute, Amsterdam, The Netherlands; 3 Cancer Nursing Research Unit (CNRU), Sydney Nursing School, University of Sydney, Sydney, Australia; 4 Breast Cancer Trials, Newcastle, Australia; 5 Department of General Practice, Faculty of Medicine, Dentistry and Health Sciences, Centre for Cancer Research, University of Melbourne, Victorian Comprehensive Cancer Centre, Melbourne, VIC Australia; 6 Centre for Online Health, The University of Queensland, St Lucia, Australia; 7 Psycho-oncology Co-operative Research Group, School of Psychology, The University of Sydney, Sydney, Australia; 8 NHMRC Clinical Trial Centre, University of Sydney, Sydney, Australia

**Keywords:** Clinical trial protocols, Patient-reported outcomes, Research waste, Education, Training

## Abstract

**Introduction:**

Failure to incorporate key patient-reported outcome (PRO) content in trial protocols affects the quality and interpretability of the collected data, contributing to research waste. Our group developed evidence-based training specifically addressing PRO components of protocols. We aimed to assess whether 2-day educational workshops improved the PRO completeness of protocols against consensus-based minimum standards provided in the SPIRIT-PRO Extension in 2018.

**Method:**

Annual workshops were conducted 2011–2017. Participants were investigators/trialists from cancer clinical trials groups. Although developed before 2018, workshops covered 15/16 SPIRIT-PRO items. Participant feedback immediately post-workshop and, retrospectively, in November 2017 was summarised descriptively. Protocols were evaluated against SPIRIT-PRO by two independent raters for *workshop protocols* (developed post-workshop by participants) and *control protocols* (contemporaneous non-workshop protocols). SPIRIT-PRO items were assessed for completeness (0 = not addressed, 10 = fully addressed). Mann–Whitney *U* tests assessed whether workshop protocols scored higher than controls by item and overall.

**Results:**

Participants (*n* = 107) evaluated the workshop positively. In 2017, 16/41 survey responders (39%) reported never applying in practice; barriers included role restrictions (14/41, 34%) and lack of time (5/41, 12%). SPIRIT-PRO overall scores did not differ between workshop (*n* = 13, median = 3.81/10, interquartile range = 3.24) and control protocols (*n* = 9, 3.51/10 (2.14)), (*p* = 0.35). Workshop protocols scored higher than controls on two items: ‘specify PRO concepts/domains’ (*p* = 0.05); ‘methods for handling missing data’ (*p* = 0.044).

**Conclusion:**

Although participants were highly satisfied with these workshops, the completeness of PRO protocol content generally did not improve. Additional knowledge translation efforts are needed to assist protocol writers address SPIRIT-PRO guidance and avoid research waste that may eventuate from sub-optimal PRO protocol content.

**Supplementary Information:**

The online version contains supplementary material available at 10.1007/s11136-022-03127-w.

## Introduction

In 2014, the Lancet launched the Reduce research Waste And Reward Diligence (REWARD) Campaign with a series of five papers that highlighted key sources of waste and inefficiency in biomedical research, recommended how to increase value and reduce waste, and proposed metrics for monitoring the implementation of these recommendations [1]. The second paper addressed waste in research design, conduct, and analysis [2]. Points raised in that paper that are addressed in this paper in relation to patient-reported outcomes (PROs) are as follows: inadequacy of study protocols, failure to involve experienced methodologists, and failure to train clinical researchers and statisticians in relevant research methods and design. Adverse consequences of not addressing these issues include gathering of inadequate or misleading information and lack of statistical precision or power. Suggested solutions included improving protocols, standardising research efforts, and training the scientific workforce.

The protocol of a clinical trial is the foundation for study planning, conduct, reporting, and appraisal [3]. It should therefore provide sufficient detail to facilitate these purposes in order to avoid wasting research resources on poorly planned, implemented, and reported trials [2, 4]. These principles apply to all trial endpoints, including PROs, which complement clinical outcomes by providing patients’ perceptions of the impact of disease and treatment. PROs include symptoms and various aspects of function (e.g. physical, emotional, social) and multi-dimensional constructs such as health-related quality of life. The importance of PROs is widely acknowledged, and PROs are commonly included in clinical trials. For example, 27% of 96,736 trials registered in ClinicalTrials.gov between 2007 and 2013 [5] and 45% of 13,666 trials registered in the Australia New Zealand Clinical Trials Registry (ANZCTR) between 2005 and 2017 [6] included one or more PROs.

Two key strategies for achieving high-quality data in clinical trials are to standardise methods for endpoint assessment across patients and sites and to minimise missing endpoint data [3]. This is particularly pertinent for PROs as they are subjective phenomena that are typically assessed repeatedly over time and cannot be retrieved retrospectively [7, 8]. It is therefore worrying that research nurses working in clinical trials report receiving insufficient information in trial protocols to implement PRO data collection consistently [9, 10], with some saying they had to revert to training received for previous trials in the absence of specific protocol instructions. Further, some noted planned PRO assessments were often missed because the protocol failed to provide contingency plans for capturing PRO data when participants missed a clinical appointment or scheduled assessment [10]. Extensive rates of avoidable missing PRO data reduce the study sample size [11, 12], risk biased and unreliable trial results, and can lead to PRO endpoints not being reported [13, 14]. This, in turn, may lessen the impact of PROs on routine clinical care, mislead clinical or health policy decision-making, reduce the value of patient participation in trials and waste limited healthcare and research resources [2, 4]. This calls into question the ethics of collecting PRO data that will not be used [15].

Systematic reviews have shown that trial protocols often lack important information regarding PROs [16–18]. This is concerning for various reasons. For example, if a protocol fails to justify the purpose of PRO assessment, or if the coverage of the PRO endpoint is poor, trial staff may perceive PROs to be less valued than other trial endpoints and invest less time and effort into ensuring high-quality PRO data collection. If there is no clear PRO research question or hypothesis, or if rates of missing PRO data are high, there may be little incentive to analyse or report PRO data in a meaningful way, again risking research waste. Indeed, there is evidence that poor PRO coverage in protocols is correlated with poor reporting of PRO results [14, 17]. To address these issues, the SPIRIT-PRO guidance was released in 2018, providing consensus-based guidance to facilitate international best practice standards for minimum PRO content in clinical trial protocols [7].

The Sydney Quality of Life Office (SQOLO) was funded by the Australian Government through Cancer Australia from 2011 to 2021 to support the national network of cancer clinical trials groups (CCTG) to include PRO endpoints in their trials/studies to international best standards. A core activity of the SQOLO was providing workshops to educate CCTG members on the scientific and logistic considerations for designing a PRO study and how these aspects should be addressed in a clinical trial protocol. A series of 2-day educational workshops was run annually 2011–2017. Although the SPIRIT-PRO guidance had not been developed at that time, members of our team (MK, RMB) were also members of the executive group that led the development of the SPIRIT-PRO guidance. In 2011, we (RMB, MK) developed the PROtocol Checklist which underpinned the content of our workshops. The PROtocol Checklist was the primary pre-cursor to the SPIRIT-PRO; it covered 13 of the 16 SPIRIT-PRO items plus several more. Workshop content covered 15 of the 16 SPIRIT-PRO checklist items.

The aim of this study was to assess whether this 2-day educational workshop directed at oncology trialists and clinician researchers from CCTGs was effective in fostering the inclusion of SPIRIT-PRO items in trial protocols. Three specific research questions were addressed: (1) Were participants satisfied with the workshop content and format? (2) Did participants use the PROtocol Checklist in the long-term? (3) Were the PRO components of protocols brought to the workshop more complete than those of contemporaneous protocols not brought to the workshop?

## Methods

### The PROtocol checklist workshop

SQOLO staff (MK, RMB, CR plus two statisticians) conducted 2-day face-to-face workshops annually between 2011 and 2017 to educate investigators and trialists about key aspects of PRO assessment within clinical trials and how to address these in protocols. Workshop format, resources, and topics covered each year are outlined in Box 1 and Online Supplement 1. Each of the 14 Australian CCTGs was invited to nominate one or more members to attend a workshop each year, and each attendee was encouraged to bring a protocol-in-development for further development. Participants who did not have a protocol-in-development could bring a finalised protocol to critically review as part of the learning process.

Prior to developing the first workshop, we developed a PROtocol Checklist to specify how PROs should be addressed in each section of a trial protocol, drawing on guidance documents and research of three key international trials groups that pioneered excellence in health-related quality of life research: the Canadian Cancer Trials Group (CCTG), the European Organisation for Research and Treatment of Cancer (EORTC), and SWOG (formerly Southwest Oncology Group). The first version of the checklist was presented in the 2011 workshop. It was updated twice to improve formatting and refine items. The third and final version (Online Supplement 2) was used in workshops 2013–2017. The PROtocol Checklist was available on the SQOLO website.

Box 1: PROtocol checklist workshops: topics, format, and resources

**Table:** 

Workshop session topics	Workshop year						
	2011	2012	2013	2014	2015	2016	2017
Introduction, rationale, objectives, and hypotheses	×	×	×	×	×	×	×
PRO measures	×	×	×	×	×	×	×
Utility measures for health economic evaluation			×	×	×	×	×
PRO questionnaire administration	×	×	×	×	×	×	×
Missing data—statistics and logistics	×	×	×	×	×	×	×
Endpoints and statistical considerations	×	×	×	×	×	×	×
Interpretation and clinical significance of PRO results				×	×	×	×
Data quality assurance, appendices, and resources	×	×	×	×			
*Workshop resources* Workshop participants were given a booklet that included the lecture slides, a bibliography of all papers referenced in the slides, excerpts from oncology protocols to illustrate how to address specific PROtocol Checklist items, and the PROtocol Checklist. See Online Supplement 1 for booklet table of contents and workshop program							
*Workshop format* The PROtocol checklist (Online Supplement 2) guided the structure of each workshop. For each topic, the general principles were explained in a lecture that included examples, either from real protocols or research publications. Each lecture was followed by a small-group discussion, facilitated by faculty, focussed on how the general principles were applied to the participants’ protocols. Workshop material was refined each year based on contemporary research and workshop participant feedback							

### Aim 1: post-workshop evaluation survey (immediate)

All participants were invited to anonymously evaluate the workshop at the end of Day 2. The survey consisted of 11 questions (Online Supplement 3) which assessed the following: usefulness of the workshop and PROtocol Checklist resource; what the most valuable topic covered was; what aspects could be improved; whether the real protocol examples provided were useful; and who from the CCTGs would benefit most from future workshops. Finally, participants were asked to provide an overall rating of the workshop on a scale of 1 (poor) to 10 (excellent).

### Aim 2: long-term research practice survey

In November 2017, all workshop participants were invited to complete an online survey via REDCap (Online Supplement 4). Respondents were asked whether and how they had used the PROtocol Checklist since they attended the workshop, whether they anticipated using it in the future, and barriers to its use.

### Aim 3: completeness of PRO content of protocols

To assess the extent to which protocols developed by participants during/after the PROtocol Checklist Workshop addressed recommended items for inclusion in PRO sections of trial protocols, as compared to a control sample of protocols, we contacted key members of the CCTGs and past workshop participants to identify and request eligible protocols. *Workshop protocols* were eligible for inclusion if they were in development when brought to the PROtocol Checklist Workshops between 2011 and 2017; were led by one of the Australian CCTGs; included a PRO endpoint; were subsequently finalised for study activation; and permission was obtained from the trial group or the trial principal investigator to include results of the protocol’s review in analyses for this paper. Eligibility criteria for *control protocols* were identical, with the exception that control protocols were only eligible for inclusion if they were *not* brought to a PROtocol Checklist Workshop (unless after being finalised) and did not receive input on development from any SQOLO staff.

Each protocol was evaluated against the full complement of items covered by the SPIRIT-PRO Checklist (Online Supplement 5) and the PROtocol Checklist by two independent trained raters ((FM or RC) and (CB, SC, DL, or JS). Raters were blinded to workshop/control status of each protocol. To ensure a standardised rating process, two experienced raters (FM, RC) developed a comprehensive rating guide for each checklist item (Online Supplement 6). All items were rated for completeness on a scale from 0 (not addressed) to 10 (fully addressed). Inter-rater reliability was assessed with weighted kappa [19]. Ratings discrepant by up to two points were averaged. Discrepancies of three or more points were discussed and resolved with MK.

### Analysis

#### Participant survey data

Workshop participants’ survey data were summarised with percentages for questions with categorical responses and means for questions with numerical responses. Free-text responses were analysed using content analysis by MT and RC.

#### Protocol evaluations

The primary analysis related to the SPIRIT-PRO checklist; PROtocol checklist items not covered by SPIRIT-PRO were addressed as a secondary analysis. An overall SPIRIT-PRO completeness score was calculated as the average of all 16 SPIRIT-PRO item scores. We calculated summary statistics of completeness scores for each SPIRIT-PRO Checklist item, the overall SPIRIT-PRO score, and each additional PROtocol Checklist item. Normality of score distributions was assessed with Kolmogorov–Smirnov tests. Differences in score distributions between control and workshop protocols were examined with Mann–Whitney *U* tests (SPSS V24) as they are robust to small sample size and non-normality. Confidence intervals of the Mann–Whitney Parameter were calculated using the R function wmwTest in the asht R package [20]. For graphical presentation, the 0 to 10 scale was categorised: 0 (not addressed), 1–3 (poorly addressed), 4–6 (acceptably addressed), 7–9 (well addressed), and 10 (fully addressed).

## Results

### Participants and post-workshop evaluation survey

From 2011 to 2017, 107 people participated in PROtocol Checklist Workshops. Participants were spread relatively evenly across years and trial groups (Table 1). All but one participant completed post-workshop evaluation surveys. All 106 respondents stated the workshop either met or exceeded their expectations, all found examples of real protocols shown during the workshop useful (2013–2017), and all indicated the PROtocol Checklist resource was useful with nearly a quarter stating they planned to use it in the future. Sessions commonly noted as most useful were as follows: PRO measures, PRO questionnaire administration, and Missing Data—Statistics and Logistics. About a third of respondents stated the sessions on statistical considerations for PROs were difficult to follow for people with no background in statistics. Some participants found the session on Utility Measures too complex and not relevant to them. Areas noted for improvement were as follows: more time needed for group discussions; provision of reading materials before the workshop to help participants prepare.

**Table 1:** Characteristics of workshop participants

	Workshop attendees *N* = 107	Invited to follow-up survey (valid email) *n* = 82	Follow-up survey *n* = 41	Time since workshop (years)	Follow-up survey non-responders *N* = 41
Year attended workshop					
2011	14	8	2	7	6
2012	14	10	4	6	6
2013	19	15	7	5	8
2014	13	11	7	4	3
2015	16	13	5	3	8
2016	15	11	7	2	4
2017	16	14	9	1	5
CTG					
AGITG	6	5	1		4
ALLG	8	7	5		2
ALTG (now TOGA)	9	9	4		5
ANZBCTG (now BCT)	7	4	4		0
ANZCHOG	4	4	2		2
ANZGOG	6	3	0		3
ANZMTG (now MASC)	16	10	2		8
ANZUP	6	6	2		4
ASSG (now ANZSA)	6	4	2		2
COGNO	8	6	4		2
PC4	11	8	5		3
PoCoG	9	8	4		4
TROG	11	8	5		3

*AGITG* Australasian Gastro-Intestinal Trials Group, *ALLG* Australasian Leukaemia and Lymphoma Group, *ALTG* Australasian Lung Cancer Trials Group, *ANZBCTG* Australian New Zealand Breast Cancer Trials Group, *ANZCHOG* Australian and New Zealand Children’s Haematology and Oncology Group, *ANZGOG* Australia New Zealand Gynaecological Oncology Group, *ANZMTG* Australia and New Zealand Melanoma Trials Group, *ANZSA* Australia and New Zealand Sarcoma Association, *ANZUP* Australian and New Zealand Urogenital and Prostate Trials Group, *ASSG* Australasian Sarcoma Study Group, *BCT* Breast Cancer Trials, *COGNO* Cooperative Trials Group for Neuro-Oncology, *MASC* Melanoma and Skin Cancer Trails Limited, *PC4* Primary Care Collaborative Cancer Clinical Trials Group, *PoCoG* Psycho-oncology Co-operative Research Group, *TOGA* Thoracic Oncology Group Australasia, *TROG* Trans-Tasman Radiation Oncology Group

Individuals perceived as being most likely to benefit from attending the workshop were protocol developers, trial coordinators, project officers, central operations staff, principal investigators, co-investigators, and research fellows. The overall rating of the workshop by participants (2013–2017, *n* = 80) ranged from 6 to 10 (where 1 = poor and 10 = excellent), with median = 9, mean = 8.6, and SD 0.96.

Written comments from respondents (Box 2) supported the results above.

Box 2: Examples of post-workshop feedback comments

**Table:** 

Typical post-workshop feedback comments
*Good comprehensive overview of QOL measures, incorporation into a protocol and other factors to consider. Good practical examples and good to be introduced to the protocol checklist*. ID72
*It was very helpful and made me feel more comfortable about including PROs in my studies*. ID16
*I will use the checklist for each new protocol and share it with principle investigators.* ID17
*The checklist is very useful*—*makes you think of things that might otherwise slip through the cracks*. ID24
Comments providing constructive criticism
*Stats session was a bit in-depth for what we would use in our roles.* ID75
*more interaction—as a lot of didactic content.* ID37

### Follow-up survey

In 2017, we attempted to contact all 107 workshop participants to complete the online follow-up survey. Many had changed workplaces and of these, we were unable to locate new contact details for 25. Of the 82 participants we successfully contacted, 41 completed the survey. Respondents were trial coordinators (4/41, 10%), principal investigators (8/41, 20%), co-investigators (8/41, 13%), CCTG protocol developers (4/41, 10%), or their roles were not specified (17/41, 41%). Based on survey responses, since attending the workshop, 23/41 (56%) reported using the checklist when developing new protocols and 10/41 (24%) when amending protocols, while 16/41 (39%) said they had not used the checklist. The majority 32/41 (78%) indicated the checklist would be useful if they were to develop a new study that included PROs.

Additional qualitative data provided as comments fell into two themes: how participants used the PROtocol Checklist since the workshop and barriers to using the PROtocol Checklist. Many of the comments supported the quantitative result noted above: over half (23/41, 56%) reported using the PROtocol Checklist when developing PRO endpoints for new trial protocols, and about a quarter noted using it to review PRO components of existing protocols (10/41, 24%). Other uses included the following: guide for what should be included in the protocol (1/41, 2.4%); integration of checklist items into the CCTG standard protocol template to improve the PRO aspects of the protocol. (1/41, 2.4%); as a reference in grant applications (2/41, 5%); and as a training resource for research assistants (1/41, 2.4%).

Regarding barriers to using the PROtocol Checklist, 8/41 (20%) participants noted not having an opportunity to use the checklist, due to either leaving their role, it not being relevant to their role, or it not being applicable to the trials they worked with. Other barriers included the following: time constraints within the busy protocol development process (4/41, 10%); forgetting to use the Checklist (3/41, 7%); finding it hard to use and the wording of some items hard to follow (2/41, 5%); not being able to find the Checklist when needed (1/41, 2.4%); protocol developers/investigators not seeing the value of adding additional detail on PROs (1/41, 2.4%); investigators wanting the protocol to be brief (1/41, 2.4%).

### Protocol evaluation

Of the 74 protocols brought to the PROtocol Checklist Workshops, 25 were finalised to study activation, of which only 16 were eligible for review. Three of these 16 protocols were already final versions, brought to the workshop by participants who did not have a protocol-in-development, to enable critical review as part of the learning process. As these three protocols could not be changed in light of workshop learnings, they were considered control protocols. A further six protocols were contributed as control protocols. Table 2 summarises the characteristics of the nine control protocols and 13 workshop protocols.

**Table 2:** Characteristics of the control and workshop protocols

	Control protocol *n* = 9	Workshop protocol *n* = 13
Date of protocol version		
2010	2 (22.2%)	0 (0%)
2011	1 (11.1%)	0 (0%)
2012	0 (0%)	0 (%)
2013	0 (0%)	2 (15.4%)
2014	0 (0%)	2 (15.4%)
2015	1 (11.1%)	2 (15.4%)
2016	1 (11.1%)	6 (46.2%)
2017	4 (44.4%)	1 (7.7%)
PRO endpoint(s)		
Primary only	2 (22.2%)	1 (7.7%)
Secondary only	5 (55.6%)	10 (76.9%)
Primary & secondary	1 (11.1%)	2 (15.4%)
Exploratory	1 (11.1%)	0 (0%)
Trial phase		
Phase I (biomed-safety)	0 (0%)	0 (0%)
Phase I (screening-feasibility)	3 (33.3%)	0 (0%)
Phase II (biomed)	3 (33.3%)	8 (61.5%)
Phase III (biomed)	2 (22.2%)	5 (38.5%)
Correlative study	1 (11.1%)	0 (0%)
Cancer type		
Brain	2 (22.2%)	1 (7.7%)
Breast	0 (0%)	1 (7.7%)
Gastro-intestinal	2 (22.2%)	0 (0%)
Leukaemia	0 (0%)	3 (23.1%)
Lung	1 (11.1%)	2 (15.4%)
Melanoma	2 (22.2%)	3 (23.1%)
Prostate	1 (11.1%)	0 (0%)
Testicular	0 (0%)	1 (7.7%)
Two or more cancer types	1 (11.1%)	2 (15.4%)
Cancer stage		
Pre cancer (at risk)	1 (11.1%)	1 (7.7%)
Early solid tumours	3 (33.3%)	3 (23.1%)
Advanced solid tumours	4 (44.4%)	6 (46.2%)
Mixed stage solid tumours	1 (11.1%)	0 (0%)
NA (blood cancers)	0 (0%)	3 (23.1%)
Healthcare stage		
Screening	2 (22.2%)	1 (7.7%)
Curative treatment	5 (55.6%)	7 (53.8%)
Palliative treatment	0 (0%)	1 (7.7%)
Psychosocial/supportive care	0 (0%)	4 (30.8%)
Follow-up	1 (11.1%)	0 (0%)
Mixed healthcare stages	1 (11.1%)	0 (0%)
Active & palliative treatment types		
Chemotherapy	1 (11.1%)	2 (15.4%)
Chemotherapy or radiotherapy	0 (0%)	1 (7.7%)
Immunotherapy	1 (11.1%)	1 (7.7%)
Surgery only	1 (11.1%)	1 (7.7%)
Surgery & chemotherapy	0 (0%)	1 (7.7%)
Surgery & radiotherapy	0 (0%)	1 (7.7%)
Targeted therapy	2 (22.2%)	2 (15.4%)
NA (other healthcare stages)	4 (44.4%)	5 (38.5%)

The 22 protocols were assessed against a total of 53 checklist sub-items that comprehensively covered both PROtocol and SPIRIT-PRO checklists. Inter-rater reliability coefficients for these are provided in Online Supplement 7. The majority were in the range considered moderate (20/53 items with weighted kappa coefficients in the range 0.41–0.60) or fair (12/53 in range 0.21–0.40) agreement [21].

Table 3 presents summary statistics for SPIRIT-PRO completeness scores for the control and workshop protocols, and Mann–Whitney *U* test results. Scores on more than 50% of the items were non-normally distributed in both groups (Kolmogorov–Smirnov p values in range < .001 to .031).

**Table 3:** Mean (M), standard deviation (SD), Median (Md), Interquartile range (IQR), Mean Rank (MR) of the completeness rating for each SPIRIT-PRO item and the total SPIRIT-PRO score for control and workshop protocols

SPIRIT-PRO item	Control protocols (*n* = 9)			Workshop protocols (*n* = 13)			Mann–Whitney test		
	*M* (SD)	Md (IQR)	MR	*M* (SD)	Md (IQR)	MR	*p* value^a,^*	95% CI^b^	Effect size^c^
SPIRIT-5a-PRO: Individual responsible	0.00 (0.00)	0.00 (0.00)	11.50	0.00 (0.00)	0.00 (0.00)	11.50	–	–	–
SPIRIT-6a-PRO: Rationale and research question	3.78 (2.59)	5.17 (4.33)	11.44	4.05 (3.62)	4.33 (6.83)	11.54	1	− 3.50; 3.33	> .001
Summarise PRO findings from past relevant studies	3.61 (3.32)	4.00 (6.00)	10.06	4.81 (4.12)	6.50 (7.50)	12.50	0.399	− 5.50; 2.99	0.04
Describe the rationale for PRO assessment	5.28 (3.49)	7.50 (5.00)	12.50	4.35 (3.64)	5.00 (7.50)	10.81	0.567	− 2.00; 4.49	0.02
Describe the PRO-specific research question	2.44 (2.20)	3.00 (2.50)	11.44	3.00 (3.55)	0.50 (5.00)	11.54	1	− 3.50; 2.50	> .001
SPIRIT-7-PRO: Objectives & hypotheses	5.06 (1.91)	5.00 (3.5)	11.44	5.08 (3.11)	5.50 (4.50)	11.54	1	− 2.50; 2.50	> .001
SPIRIT-10-PRO: Eligibility criteria	4.89 (5.01)	4.00 (10.00)	10.67	6.15 (5.06)	10.00 (10.00)	12.08	0.594	− 9.99; 0.00	0.02
Specify any PRO-specific eligibility criteria	4.89 (5.01)	4.00 (10.00)	10.67	6.15 (5.06)	10.00 (10.00)	12.08	0.594	− 9.99; 0.00	0.02
If PROs will not be collected in the entire study sample, provide a rationale and describe the method for obtaining the PRO subsample Note. N/A is an option	NA	NA	NA	NA	NA	NA	–	–	–
SPIRIT-12-PRO:Concepts & domains	2.24 (2.20)	1.33 (2.67)	8.22	4.73 (3.15)	4.00 (5.17)	13.77	0.052*	− 4.83; 0.00	0.19
Specify the PRO concepts/domains used to evaluate the intervention	3.39 (2.51)	3.00 (3.50)	8.94	5.65 (3.18)	6.00 (2.00)	13.27	0.131	− 4.99; 0.99	0.11
For each of the PRO concepts or domains used to evaluate the intervention, specify the analysis metric	1.78 (2.81)	0.50 (1.00)	7.78	4.81 (3.39)	4.00 (6.00)	14.08	0.026*	− 5.00; − 0.49	0.24
For each of the PRO concepts or domains used to evaluate the intervention, specify the principal time point or period of interest	1.56 (2.52)	0.00 (1.50)	8.94	3.73 (3.63)	4.00 (4.50)	13.27	0.123	− 4.99; 0.00	0.12
SPIRIT-13-PRO:Participant timeline	3.63 (1.15)	3.82 (0.58)	10.94	4.16 (1.88)	3.83 (2.67)	11.88	0.763	− 2.08; 1.08	0.01
Include a schedule of PRO assessments, specifying which measures will be used at each assessment	7.28 (3.60)	9.50 (5.00)	9.89	8.92 (2.21)	10.00 (1.50)	12.62	0.307	− 4.50; 0.00	0.05
Provide a rationale for the assessment time points	1.22 (1.39)	0.50 (1.00)	10.89	2.27 (2.67)	1.00 (5.00)	11.92	0.733	− 3.50; 0.50	0.01
Is initial PRO assessment pre-randomisation? If initial PRO assessment is post-randomisation provide a justification	10.00 (0.0)	10.00 (0.0)	12.00	5.42 (4.98)	7.50 (10)	7.75	0.070	–	0.17
Specify PRO assessment time windows	3.06 (3.40)	2.00 (3.00)	9.89	4.54 (3.76)	4.00 (6.50)	12.62	0.345	− 4.99; 1.49	0.05
Specify whether PRO collection is prior to clinical assessments	3.00 (4.80)	0.00 (5.50)	10.14	2.73 (4.67)	0.00 (5.00)	9.09	0.661	− 0.00; 1.00	0.01
If using multiple questionnaires, specify whether order of administration will be standardised. Note N/A is an option	0.00 (0.00)	0.00 (0.00)	9.50	0.00 (0.00)	0.00 (0.00)	9.50	–	–	–
SPIRIT-14-PRO: Sample size	3.72 (4.35)	1.50 (7.00)	11.28	4.00 (4.95)	0.50 (10.00)	11.65	0.914	− 4.99; 4.99	.001
SPIRIT-18a(i)-PRO: PRO instrument	2.62 (1.79)	2.75 (1.00)	9.28	3.63 (1.77)	3.88 (2.50)	13.04	0.192	− 2.75; 0.74	0.09
Justify the PRO instrument to be used	3.72 (2.32)	4.00 (1.50)	9.83	5.04 (2.93)	5.00 (4.50)	12.65	0.331	− 3.99; 1.50	0.05
Describe the PRO instrument in terms of domains, number of items, recall period, instrument scaling/scoring	3.56 (1.65)	3.50 (1.00)	10.28	4.69 (3.09)	5.50 (3.50)	13.35	0.481	− 3.49; 1.49	0.03
Evidence of PRO instrument measurement properties, interpretation guidelines, and patient acceptability/burden should be provided or cited if available, ideally in the population of interest	1.61 (1.87)	1.00 (2.00)	9.89	2.73 (2.60)	1.50 (4.50)	12.62	0.346	− 3.50; 0.50	0.05
State whether the measure will be used in accordance with any user manual and specify and justify deviations if planned	1.61 (3.20)	0.00 (0.00)	10.56	2.08 (3.59)	0.00 (2.00)	12.15	0.518	− 1.99; 0.00	0.02
SPIRIT-18a(ii)-PRO: Mode & setting of administration	6.33 (3.63)	7.24 (4.50)	12.61	5.25 (4.28)	5.00 (8.75)	10.73	0.522	− 2.75; 5.00	0.02
Include a data collection plan outlining the permitted mode(s) of administration	4.89 (4.20)	4.50 (7.50)	12.28	4.35 (4.36)	3.00 (9.00)	10.96	0.658	− 3.00; 4.50	0.01
Specify PRO data collection setting (e.g. clinic, home, other)	7.78 (4.41)	10.00 (0.00)	12.56	6.15 (5.06)	10.00 (10.00)	10.77	0.457	− 0.00; 9.00	0.03
SPIRIT-18a(iii)-PRO: Translations	2.53 (2.52)	2.50 (5.00)	11.00	3.42 (3.98)	0.50 (8.00)	11.85	0.781	− 4.75; 2.50	0.004
Specify whether more than one language version will be used	2.56 (2.49)	2.50 (5.0)	11.06	3.42 (3.98)	0.50 (8.00)	11.81	0.808	− 4.50; 2.50	0.004
If a translation will be used, state whether it was developed using currently recommended methods Note. N/A is an option	0.00^d^ (0.00)	0.00^d^ (0.00)	–	–	NA	NA	–	–	–
SPIRIT-18a(iv)-PRO: Proxy assessment	8.00^d^ (0.00)	8.00^d^ (0.00)	–	6.00^d^ (0.00)	6.00^d^ (0.00)	–	–	–	–
SPIRIT-18b(i)-PRO:Strategies for minimising missing data	1.72 (2.02)	1.00 (4.00)	11.28	2.35 (3.27)	0.50 (4.00)	11.65	0.918	− 2.99; 1.00	0.001
SPIRIT-18b(ii)-PRO:Discontinuation or withdrawal	4.33 (2.93)	5.00 (4.50)	10.00	5.58 (3.11)	5.00 (4.00)	12.54	0.384	− 4.00; 1.99	0.04
SPIRIT-20a-PRO: PRO analysis methods	2.82 (2.85)	2.50 (2.75)	11.00	5.19 (3.30)	4.50 (6.00)	11.85	0.070	− 5.49; 0.25	0.004
State PRO analysis methods	4.56 (3.40)	5.00 (5.50)	8.28	7.31 (2.90)	8.00 (3.50)	13.73	0.055*	− 6.49; 0.00	0.18
State any plans for addressing multiplicity or type 1 (α) error	1.11 (3.33)	0.00 (0.00)	10.22	3.08 (4.80)	0.00 (10.00)	12.38	0.312	− 9.99; 0.00	0.05
SPIRIT-20c-PRO: Missing data	0.08 (0.25)	0.00 (0.00)	10.22	1.29 (1.70)	0.25 (2.50)	12.38	0.044*	− 2.49; 0.00	0.05
State how missing data will be described	0.00 (0.00)	0.00 (0.00)	11.50	0.00 (0.00)	0.00 (0.00)	11.50	–	–	–
Outline the methods for handling missing items and entire assessments (e.g. approach to imputation and sensitivity analyses)	0.167 (0.50)	0.00 (0.00)	8.56	2.58 (3.40)	0.50 (5.00)	13.54	0.044*	− 4.99; 0.00	0.20
SPIRIT-22-PRO: Harms	0.741 (2.22)	0.00 (0.00)	10.61	2.05 (3.98)	0.00 0.00)	12.12	0.456	− 4.49; 4.55	0.03
State whether or not PRO data will be monitored during the study to inform the clinical care of individual trial participants	1.11 (3.33)	0.00 (0.00)	10.72	2.31 (4.39)	0.00 (0.00)	12.04	0.516	− 0.00; 0.00	0.02
If PRO data will be monitored during the study to inform clinical care of individual participants, state how this will be managed in a standardised way Note. N/A is an option	0.00^d^ (0.00)	0.00^d^ (0.00)	–	10.00^d^ (0.00)	10.00^d^ (0.00)	–	–	–	–
If PRO data will be monitored during the study to inform clinical care of individual participants, describe how this process will be explained to participants e.g. in the participant information sheet and consent form. Note. N/A is an option	10.00^d^ (0.00)	10.00^d^ (0.00)	–	6.67^e^ (5.77)	10.00^e^ (0.00)	–	–	–	–
Total SPIRIT-PRO Score	3.00 (1.36)	3.51 (2.14)	9.89	3.79 (2.06)	3.81 (3.24)	12.62	0.349	− 2.67; 1.47	0.01

For completeness rating scores, higher scores indicate more completely addressed items

^a^Mann–Whitney *U* tests, * indicates statistical significant at 95% level (*p* < 0.05)

^b^Confidence intervals of the Mann–Whitney parameter can be interpreted as confidence intervals on the difference in medians. Some 95% confidence limits were very close to zero; the smallest of these was − 0.00004 and the largest was 0.00007

^c^Effect sizes of the Mann–Whitney parameter

^d^Based on *n* = 1

^e^Based on *n* = 3

The median (M) SPIRIT-PRO total score for workshop protocols was *M*_W_ = 3.81/10 (interquartile range (IQR_W_) = 3.24) versus *M*_C_ = 3.51/10 (IQR_C_ = 2.14) for control protocols (*p* = 0.35). Despite no difference in overall scores, there were some item-level differences. For SPIRIT-12-PRO: ‘*Specify the concepts/ domains used to evaluate the intervention’*, workshop protocols were more complete than control protocols (*M*_W_ = 4.00 (IQR_W_ = 5.17), *M*_C_ = 1.33 (IQR_C_ = 2.67), *p* = 0.052). Analysis of the three subcomponents of this item revealed that this difference was largely driven by the workshop protocols being more complete than control protocols on specifying the analysis metric for each PRO concept or domain used to evaluate the intervention (*M*_W_ = 4.00 (IQR_W_ = 6.00), *M*_C_ = 0.50 (IQR_C_ = 1.00), *p* = 0.026).

Workshop protocols were also more complete than control protocols on stating PRO analysis methods (SPIRIT-20a-PRO subcomponent: *M*_W_ = 8.00 (IQR_W_ = 3.50), *M*_C_ = 5.00 (IQR_C_ = 5.50), *p* = 0.055) and SPIRIT-20c-PRO item ‘*Missing data’* (*M*_W_ = 0.25 (IQR_W_ = 2.50), *M*_C_ = 0.00 (IQR_C_ = 0.50), *p* = 0.044), due entirely to the subcomponent about outlining methods for handling missing data (*M*_W_ = 0.50 (IQR_W_ = 5.00), *M*_C_ = 0.00 (IQR_C_ = 0.50), *p* = 0.044). Of note, none of the protocols addressed SPIRIT-5a-PRO: ‘*specifying the individual responsible for the PRO content’*, or SPIRIT-20c-PRO: ‘*state how missing data will be described’*.

**Fig. 1:**
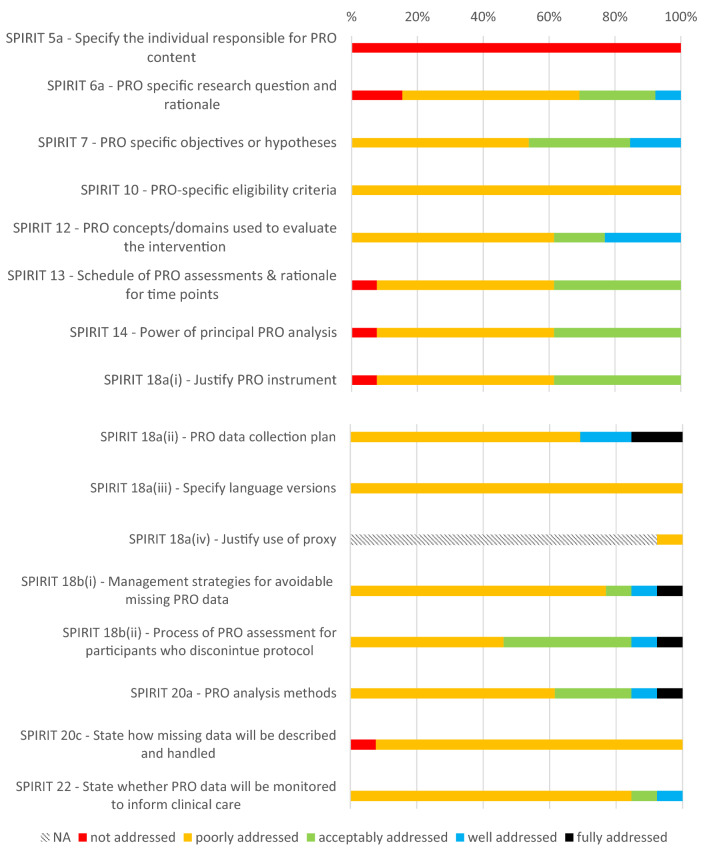
SPIRIT-PRO items addressed in protocols developed by workshop participants (*n* = 13)

Figure 1 shows completeness ratings of the SPIRIT-PRO items in the 16 protocols developed by workshop participants. Most items were predominantly poorly addressed and very few items were fully addressed. Only two items were at least 50% acceptably or well addressed.

Table 4 presents summary statistics for completeness scores on the 18 additional PROtocol Checklist items for the control and workshop protocols, and Mann–Whitney *U* test results. The workshop protocols were more complete than control protocols on describing methods for deriving PRO endpoints from PRO data (*M*_W_ = 4.23/10 (IQR_W_ = 3.46), *M*_C_ = 1.28/10 (IQR_C_ = 1.75), *p* = 0.039). However, control protocols were more complete than workshop protocols on providing sample patient information sheet and consent forms about PRO assessment and access to PRO data (*M*_W_ = 0/10 (IQR_W_ = 0.00), *M*_C_ = 0/10 (IQR_C_ = 10.00), *p* = 0.035), noting that the median was zero (‘not addressed’) for both groups. The mean completeness scores for workshop protocols were higher than those of the control protocols on nine of the 18 additional PROtocol Checklist items, but the score distributions did not differ significantly.

**Table 4:** Mean (M) and standard deviation (SD), Median (Md), Interquartile range (IQR), Mean Rank (MR) of the completeness rating for each additional PROtocol Checklist for control and workshop protocols

PROtocol checklist item #	Additional items	Control protocols (*n* = 9)			Workshop protocols (*n* = 13)			Mann–Whitney test		
		M (SD)	Md (IQR)	MR	M (SD)	Md (IQR)	MR	p value^a^,*	95% CI^b^	Effect size^c^
2	In the protocol summary—Identify specific PRO endpoint(s), specifying key PRO construct(s)/domain(s), time point(s), analysis metric(s). (i.e. change in score)	2.39 (2.04)	2.00 (3.00)	10.17	3.46 (2.90)	2.00 (3.50)	12.42	0.439	− 3.49; 1.49	0.03
3	In the protocol summary—PRO assessment included in the study schema/assessment schedule	6.67 (5.00)	10.00 (10.00)	12.67	5.00 (5.00)	5.00 (10.00)	10.69	0.447	− 0.00; 9.99	0.03
4^d^	Do the stated PRO objectives/hypotheses include time points?	0.22 (0.51)	0.00 (0.00)	9.89	1.58 (2.33)	0.00 (3.50)	12.62	0.258	− 3.49; 0.00	0.07
13	Specify who is responsible for delivering PRO questionnaires to patients and retrieving completed questionnaires from them, or if online, who is responsible for sending reminders	1.83 (2.45)	0.50 (4.00)	11.11	1.92 (2.95)	1.00 (1.00)	11.77	0.834	− 1.00; 2.99	0.003
15	Specify what should be done when PRO assessments are missed, including contingency plans for following up patients who miss PRO assessments and who is responsible for implementing them	2.61 (3.43)	1.00 (3.50)	11.17	3.31 (3.57)	1.00 (6.50)	11.73	0.865	− 4.99; 1.49	0.002
24	Specify where PRO questionnaire data will be stored	7.28 (4.41)	10.00 (0.00)	11.56	7.69 (3.29)	10.00 (0.00)	11.56	1	− 0.00; 0.00	< 0.001
25	Specify security measures in place to ensure confidentiality of patient data	6.83 (3.05)	8.00 (3.50)	12.44	6.77 (2.31)	7.00 (2.00)	10.85	0.591	− 2.50; 2.50	0.02
26	Specify what will happen to a patient's PRO data if that patient decides to exit the study	1.11 (3.33)	0.00 (0.00)	10.72	2.31 (4.39)	0.00 (0.00)	12.04	0.516	− 0.00; 0.00	0.02
27^d^	Describe methods for deriving PRO endpoints from PRO data	1.28 (1.75)	0.50 (2.00)	8.06	4.23 (3.46)	4.00 (5.50)	13.88	0.039*	− 5.99; − 0.00	0.21
29	Where possible, reference scoring manuals for summated scales from questionnaires (domain-specific &/or total), and methodological papers for composite endpoints (e.g. QTWiST)	1.11 (3.33)	0.00 (0.00)	11.22	1.54 (1.04)	0.00 (0.00)	11.69	0.822	− 0.00; 0.00	0.003
30	Describe PRO responder definitions (size and duration of benefit), where relevant	0.00 (0.00)	0.00 (0.00)	5.50	1.71 (3.40)	0.00 (3.00)	7.21	0.261	− 3.00; 0.00	0.07
32	State minimal important difference (with reference/s)—relevant to sample size calculations, responder definitions, and interpreting clinical significance of results	0.00 (0.00)	0.00 (0.00)	10.00	2.31 (4.39)	0.00 (0.00)	12.54	0.144	− 0.00; 0.00	0.11
35	Provide references for what is known about PROs (as per Background and Rationale section)	7.78 (4.41)	10.00 (0.00)	12.94	5.46 (5.11)	10.00 (10.00)	10.50	0.322	− 0.00; 9.99	0.07
36	Provide references for PRO data analyses and methods for handling missing data	0.00 (0.00)	0.00 (0.00)	10.50	1.54 (3.76)	0.00 (0.00)	12.10	0.255	− 0.00; 0.00	0.05
37	Provide copies of PRO questionnaires	7.78 (4.4)	10.00 (0.00)	13.06	5.38 (5.19)	10.00 (10.00)	10.42	0.279	− 0.00; 9.99	0.06
38	Provide evidence of permission to use PRO questionnaires (if permission not required, this is stated)	1.11 (3.33)	0.00 (0.00)	11.72	0.769 (2.77)	0.00 (0.00)	11.35	0.841	− 0.00; 0.00	0.003
39	Provide copies of the Patient-Reported Outcomes (PRO) Completion and Missing Data (CoMiDa) Form—to record reasons for missing PRO data, which may inform analyses	0.00 (0.00)	0.00 (0.00)	11.00	0.769 (2.77)	0.00 (0.00)	11.85	0.459	− 0.00; 0.00	0.03
40	Provide sample Patient Information Sheet and Consent form (in which the patient is informed about the requirement and purpose of PRO questionnaires in this research, who has access to the PRO data and who to contact with questions)	4.44 (5.27)	0.00 (10.00)	14.11	0.39 (1.39)	0.00 (0.00)	6.69	0.035*	− 0.00; 10.00	0.22

For completeness rating scores, ﻿higher scores indicate more completely addressed items

^a^Mann–Whitney *U* tests, * indicates statistical significant at 95% level (p < 0.05)

^b^Confidence intervals of the Mann–Whitney parameter can be interpreted as confidence intervals on the difference in medians. Some 95% confidence limits were very close to zero; the smallest of these was − 0.0001 and the largest was 0.00008

^c^Effect sizes of the Mann–Whitney parameter

^d^These represent part of PROtocol Checklist items 4 and 27; the other parts coincided with SPIRIT-PRO items 7 and 12_ii_y

## Discussion

We provided a 2-day PROtocol Checklist workshop designed to equip trialists with the motivation, knowledge, and resources to write PRO content in trial protocols. Participants rated workshops highly and acknowledged the value of PROs and the PROtocol checklist. However, few reported using the PROtocol checklist in the long term; barriers included staff churn and time constraints. PRO components of workshop protocols were generally not more complete than control protocols, and were often incomplete.

The specific protocol omissions we identified potentially increase the risk of research waste in the following ways. No protocol identified an individual responsible for PROs, increasing the risk of the PROs not being analysed and reported adequately/at all; this relates to the ‘failure to involve experienced methodologists’ source of research waste [2]. Failure to report PRO data is concerningly common, with rates ranging from 37 to 43% in four reviews [22–25]. Multiplicity was poorly addressed; this leads to a higher chance of false-positive findings, potentially misleading clinical practice and policy [26]. PRO assessment time points were often not specified, with staff at recruiting sites not knowing when to collect data, increasing the risk of missing PRO data [9, 10, 12]. Few protocols explained how to handle missing data, which could lead to inappropriate handling of missing data and bias [12, 27]. The general inadequacy of PRO content suggests PRO endpoints were not prioritised by the trials groups, staff or principal investigators, so site staff training and PRO analysis may not have been appropriately budgeted for, reducing the quality of PRO data collected and the likelihood of publication. Similar deficiencies and risks have been identified in previous systematic reviews of trial protocols [16, 18].

We did not obtain all protocols brought to the workshop, so our results may not be representative. Nevertheless, our findings were disappointing after nearly a decade of delivering workshops specifically tailored to the topic and receiving glowing participant evaluations. How might future educational efforts be more effective?

First, target audience: We encouraged CCTGs to send principal investigators and trial group staff with protocol development roles to our workshops. Trial staff may not have felt they had the authority to make substantial changes to protocols, in which case the attitudes of principal investigators and trial staff managers about PRO endpoints and PRO-specific protocol content would be critical. It is therefore concerning that oncology trialists and principal investigators have expressed skepticism about PROs due to their subjectivity and focus on survival outcomes, relegating PROs to a relatively low position in the trial outcome hierarchy [28, 29]. Our long-term survey revealed two challenges to building PRO expertise within trials groups: we were unable to contact about 20% of workshop participants, and 34% of responders said their current roles did not involve developing protocol content. Staff turnover and role change is inevitable, so ongoing workforce training is needed.

Second, workshop format and instructional methods: Long-term learning is facilitated by a series of temporally separated lessons with practice opportunities distributed within and across lessons—the ‘spacing effect’ [30]. Our 2-day intensive format was internally spaced; content was organised into a series of topics, with lectures punctuated by periods of reflection (small-group discussion) and practice (individual protocol writing). Worked examples are recommended to illustrate underlying principles, especially for novice learners [30]. We included excerpts from oncology protocols to illustrate how to address PRO issues, but learning would have been bolstered by ‘faded’ (partially complete) examples and comparison among poor versus good examples, plus practice exercises/questions for each protocol topic [30]. For questions, explanatory feedback about why an answer is correct/incorrect is an important instructional method. Finally, novice learners are more subject to cognitive overload than experienced learners and may benefit from different instructional methods [30]. Few of our workshop participants had previous training in PROs, and immediately post-workshop, participants could have had competing work priorities. Without repetition and reinforcement of the knowledge gained during the workshop, participants would likely have forgotten the key principles and learnings.

Solving this array of issues requires a multifactorial response. Greater appreciation of the value of PROs by investigators, trial group managers, and staff may motivate more investment in developing PRO expertise and prioritising PRO content during protocol development. Educational resources are needed, tailored to the training needs of various target audiences, using evidence-based instructional methods and formats. Self-directed online modular formats provide scheduling flexibility for busy professionals. Stand-alone courses could be provided by organisations like PRAXIS Australia [31] and Cancer Institute New South Wales [32], or provided by universities as part of graduate and post-graduate level courses. Professional development incentives may be effective for short-term engagement but not necessarily for deep learning and sustained practice. An interesting new adjunct to training currently in development is an online protocol authoring tool based on SPIRIT, SEPTRE, designed to help develop protocol content, with future plans to incorporate SPIRIT-PRO guidance [33]. Trials groups could be encouraged to incorporate SPIRIT-PRO into their protocol templates, and trial registries and journals could require authors to comply with SPIRIT and SPIRIT-PRO. Trials groups could encourage investigators to consider PROs early in the development process, before drafting the protocol, and encourage statisticians to consider PROs using the SISAQOL guidance [26] when writing the statistical analysis plan. Again, guidance alone does not ensure compliance; motivation, training, and expertise are also needed.

Why were there so few differences between the workshop and control protocols? One possible explanation is that workshops were not effective, as discussed above. Another is contamination effect: we opted for contemporaneous controls rather than historical controls, so control protocols were developed during the period when workshops were being conducted, and may have been influenced indirectly by knowledge developed by CCTG staff who attended a workshop and were responsible for protocol writing for their group. Also, the PROtocol Checklist was promoted as a resource to the CCTGs and freely available via the SQOLO website. It is interesting that even though about a third of workshop respondents stated that the sessions on statistical considerations for PROs were difficult to follow for people without statistical backgrounds, the subcomponent ‘*state the PRO analysis methods*’ of SPIRIT-20a-PRO (*PRO analysis methods*) improved in completeness relative to controls, suggesting the workshop was effective on that point.

Our study had limitations. An a priori target sample size calculation was not useful as our access to protocols was capped, and post hoc power analyses are circular in reasoning because they are largely a function of the data obtained [34]. Despite a relatively large number of participants attending the workshops, we were able to obtain only a small sample of protocols, particularly control protocols. This was a major limitation, both in terms of the play of chance in which protocols we sampled and power to detect differences due to the workshops. Availability was limited: of 74 protocols brought to the workshops, only 25 were finalised to study activation, nine of which were excluded because QOL Office staff had contributed to them. Confidentiality concerns may have limited the availability of protocols, including control protocols. There may also have been some selection bias if CCTGs offered high-quality protocols as controls. Greater balance in study characteristics between workshop and control protocols would have been preferable, but was not possible given the constraints of our pragmatic sample accumulation. It is unclear how the imbalances influenced our findings, but we note that the SPIRIT-PRO guidance is applicable to all phases, endpoints, and healthcare settings. One-third of the controls were led by one investigator and trials group, which may have limited the variability of the control group and potentially compromised the independence of the observations within the control group. The workshops were based on the SQOLO PROtocol Checklist but we focussed our evaluation on SPIRIT-PRO because SPIRIT-PRO has more relevance to the trials community moving forward, and it was possible to do so because our workshop covered 15/16 SPIRIT-PRO items. We did not assess workshop participant or CCTG attitudes towards the value of PRO assessment, or their training or experience with PROs, so we could not assess whether this was an underlying influence on how much attention PROs were given in protocols. It would also have been useful to know this about the developers of the control protocols. Finally, we did not assess supporting documents, such as site manuals, standard operating procedures, or statistical analysis plans, all of which may have contained information on PRO endpoints to complement the protocol. These supplementary documents are difficult to obtain and there is huge variation in how different recruiting sites and trials groups manage such instructions, particularly those relating to the conduct of the trial. These limitations suggest improvements for future studies with similar aims.

## Conclusions/implications

Writing specialist PRO content for trial protocols requires expertise, time, and effort if PRO content of protocols is to meet even the minimum standards set out in the SPIRIT-PRO guidance. This requires two things: appreciation by clinician researchers and trials group managers of the value of PRO endpoints in clinical trials, and investment in effective workforce training. One-off educational workshops are not enough to develop needed expertise in PROs. A series of easily accessible and effective educational activities, longer-term mentoring programs, and institutional requirements for use of existing resources may improve the PRO content of trial protocols and PRO study design, which in turn would likely improve PRO conduct and reporting, all reducing research waste associated with these aspects of clinical research.

## Supplementary Information

Below is the link to the electronic supplementary material.

Supplementary file1 (PDF 996 kb)

Supplementary file2 (PDF 440 kb)

Supplementary file3 (PDF 226 kb)

Supplementary file4 (PDF 194 kb)

Supplementary file5 (PDF 278 kb)

Supplementary file6 (PDF 286 kb)

Supplementary file7 (PDF 242 kb)
