# Supplementary material for: Improving the patient-reported outcome sections of clinical trial protocols: a mixed methods evaluation of educational workshops
Source: Qual Life Res. 2022 May 12;31(10):2901–16. doi: 10.1007/s11136-022-03127-w (PMC9470723; doi:10.1007/s11136-022-03127-w)
Supplement: Supplementary file 1 — Supplementary file1 (PDF 996 kb) [file 11136_2022_3127_MOESM1_ESM.pdf]

Improving the patient-reported outcome sections of clinical trial protocols: a mixed methods evaluation of educational workshops

Madeleine T King[1*], Margaret-Ann Tait[1], Rachel Campbell[1], Fabiola Müller[1,2], Claudia Rutherford[1,3], Corinna Beckmore[4], Sophie Chima[5], Danette Langbecker[6], Joanne Shaw[7], Rebecca Mercieca-Bebber[8]

1. University of Sydney, School of Psychology, Sydney Quality of Life Office, Australia
2. Department of Medical Psychology, Amsterdam University Medical Centers, University of Amsterdam, Amsterdam Public Health Research Institute, Amsterdam, the Netherlands
3. University of Sydney, Sydney Nursing School, Cancer Nursing Research Unit (CNRU), Sydney, Australia
4. Breast Cancer Trials, Newcastle, Australia
5. Department of General Practice, Faculty of Medicine, Dentistry and Health Sciences, Centre for Cancer Research, University of Melbourne, Victorian Comprehensive Cancer Centre, Melbourne, Vic., Australia.
6. Centre for Online Health, The University of Queensland, St Lucia, Australia
7. The University of Sydney, School of Psychology, Psycho-oncology Co-operative Research Group, Sydney Australia
8. University of Sydney, NHMRC Clinical Trial Centre, Sydney, Australia

*madeleine.king@sydney.edu.au

Online supplement 1: Workshop booklet table of contents and program

PROtocol Checklist Workshop
Quality of Life Office

University of Sydney
29-30 May 2014

Workshop Booklet

THE UNIVERSITY OF SYDNEY

Australian Government
Cancer Australia

PoCoG

Table of Contents

Section	Page number
**Program Outline**	4
**Biographies for Staff**	6
**Session 1: Introduction, Rationale, Objectives and Hypotheses**	8
**Session 2: PRO measures**	22
**Session 3: Utility measures**	47
**Session 4: PRO questionnaire administration**	59
**Session 5: Missing data**	77
**Session 6: Endpoints and Statistical Considerations**	94
**Session 7: Interpretation and Clinical significance of PRO results**	109
**Session 8: Quality assurance, appendices and resources**	116
**PRO Excerpts - PLUNG protocol** (shared in confidence)	126
**- HPV protocol** (shared in confidence)	142
**- OUTBACK protocol** (shared in confidence)	156
**PROtocol Checklist**	160
**PRO Completion and Missing Data (CoMiDa) Form**	162
**Participant List**	165
**References**	166

Program Outline – Day 1

Thursday 29 May 2014

Time	Session	Speaker
10.30am	Registration and Morning Tea	
**10.45**	**Session 1: Introduction, rationale, objectives and hypotheses**<br>Welcome, introductions and house-keeping<br>Patient-Reported Outcomes (PROs) terminology and conceptual model<br>Importance of the protocol<br>Introducing the PROtocol Checklist –plus workshop format and overview<br>The Protocol: Background and Rationale<br>The Protocol: Objectives and Hypotheses<br>Reflect, review, write – small groups or individually	MK
12.30pm	Lunch	
**1.00**	**Session 2: PRO measures**<br>Available PRO questionnaires – what's out there<br>Choosing the best PRO questionnaire(s) to match your Objectives/Hypotheses<br>Psychometric properties required<br>Details of PRO questionnaires to include in your protocol<br>Reflect, review, write – small groups or individually	MK and RMB
3.15	Afternoon tea	
**3.30**	**Session 3: Utility measures**<br>Economic evaluation and cost utility analysis (CUA) in clinical trials<br>HRQoL, health utilities and QALYs<br>Commonly used valuation methods – SG, TTO, DCE<br>Multi-attribute utility instruments (MAUIs, e.g. EQ-5D, AQOL)	RV - CREST
5.00	**Plenary discussion: Day 1 Q&A**	ALL
5.30	End of Day 1	

Program Outline – Day 2

Friday 30 May 2014

Time	Session	Speaker
**9.00am**	**Session 4: PRO questionnaire administration**<br>Inclusion and exclusion criteria specific to PRO assessment<br>Administration of PRO questionnaires – place and mode of administration<br>Timing of PRO assessments<br>Reflect, review, write – small groups or individually	MK
11.00	Morning Tea	
**11.15**	**Session 5: Missing data - statistics and logistics**<br>The effects of missing data on power<br>The effects of missing data on bias<br>Types of missingness<br>Collection of auxiliary data<br>Elements of prevention<br>Reflect, review, write – small groups or individually	DC and MK
1.00pm	Lunch	
**1.30**	**Session 6: Endpoints and Statistical considerations**<br>Endpoints<br>• Questionnaire scoring<br>• Methods for deriving PRO endpoints<br>• Analysis metric<br>• Responder definitions<br>Statistical considerations<br>• Power/sample size calculations<br>• Minimal important difference<br>• Statistical analysis<br>• Assessment of missing data<br>• Handling missing data<br>• Multiple comparisons<br>Reflect, review, write – small groups or individually	DC
3.00	Afternoon tea	
**3.15**	**Session 7: Interpretation and Clinical significance of PRO results**<br>Why PRO results are challenging to interpret<br>Aids to interpretation - Effect sizes, minimally important difference & clinical anchors	MK
**3.50**	**Session 8: Quality assurance, appendices and resources**<br>How to get the best quality data – tips and resources	RMB
**4.10**	**Plenary discussion: Day 2 Q&A**<br>Workshop evaluation	ALL
4.30	End of Day 2	
