# Supplementary material for: Improving the patient-reported outcome sections of clinical trial protocols: a mixed methods evaluation of educational workshops
Source: Qual Life Res. 2022 May 12;31(10):2901–16. doi: 10.1007/s11136-022-03127-w (PMC9470723; doi:10.1007/s11136-022-03127-w)
Supplement: Supplementary file 2 — Supplementary file2 (PDF 440 kb) [file 11136_2022_3127_MOESM2_ESM.pdf]

Improving the patient-reported outcome sections of clinical trial protocols: a mixed methods evaluation of educational workshops

Madeleine T King[1*], Margaret-Ann Tait[1], Rachel Campbell[1], Fabiola Müller[1,2], Claudia Rutherford[1,3], Corinna Beckmore[4], Sophie Chima[5], Danette Langbecker[6], Joanne Shaw[7], Rebecca Mercieca-Bebber[8]

1. University of Sydney, School of Psychology, Sydney Quality of Life Office, Australia
2. Department of Medical Psychology, Amsterdam University Medical Centers, University of Amsterdam, Amsterdam Public Health Research Institute, Amsterdam, the Netherlands
3. University of Sydney, Sydney Nursing School, Cancer Nursing Research Unit (CNRU), Sydney, Australia
4. Breast Cancer Trials, Newcastle, Australia
5. Department of General Practice, Faculty of Medicine, Dentistry and Health Sciences, Centre for Cancer Research, University of Melbourne, Victorian Comprehensive Cancer Centre, Melbourne, Vic., Australia.
6. Centre for Online Health, The University of Queensland, St Lucia, Australia
7. The University of Sydney, School of Psychology, Psycho-oncology Co-operative Research Group, Sydney Australia
8. University of Sydney, NHMRC Clinical Trial Centre, Sydney, Australia

*madeleine.king@sydney.edu.au

Online supplement 2: The PROtocol Checklist

Quality of Life Office

**PROtocol Checklist:**<br>An expanded checklist for research protocols including Patient-Reported Outcomes (PROs), including health-related quality of life (HRQoL)	**Included Y/N**	**Specify section and page number**	**Comments**
**OVERALL COMMENTS**			
**LIST OF COLLABORATORS**<br>1. Name the person responsible for the PRO components of the protocol			
**PROTOCOL SUMMARY**<br>2. Identify specific PRO endpoint(s), specifying key PRO construct(s)/domain(s), time-point(s), analysis metric(s)<br>3. PRO assessment included in the study schema / assessment schedule			
**OBJECTIVES and/or HYPOTHESES**<br>4. Identify the PRO objective(s) and/or hypotheses – specifying the key PRO domains and time-points.			
**BACKGROUND and RATIONALE**<br>5. Describe what is currently known about this/these PRO/s in this/relevant patient populations and interventions, and the gaps in current literature.<br>6. Provide a clinical rationale for PRO measurement in this setting – e.g. superior intervention/ negative impact of intervention<br>7. Describe the PRO constructs used to evaluate the intervention – e.g. specific symptom(s), specific domain(s) of HRQoL, overall HRQoL.			
**METHODS**<br>*More detail on PRO assessment may be provided in Standard Operating Procedures (SOPs) and/or the site/operations manual. Refer to additional guidance documentation (relevant to PRO assessment) when necessary.*<br>**<u>Inclusion/exclusion criteria specific to PRO assessment:</u>**<br>8. In addition to criteria for inclusion in the whole project, specific PRO inclusion/exclusion criteria related to language spoken or literacy may be required.<br>**<u>Questionnaires:</u>**<br>9. Specify which PRO questionnaire/s will be used, and justify by linking choice to specific domains specified in PRO objectives/hypotheses.<br>10. Describe the questionnaire (number of questions, response scale, how it is scored [domain/total score], score range, estimated time to complete, etc)<br>11. Provide citations for papers that describe the validity, reliability and responsiveness of the questionnaire.<br><br>**<u>Administration:</u>**<br>12. Specify how PRO will be assessed - pencil and paper, online, etc.			

13. Specify who is responsible for delivering PRO questionnaires to patients and retrieving completed questionnaires from them, or if online, who is responsible for sending reminders.<br>14. Specify where PRO will be assessed - clinic, home, etc.<br>15. Specify what should be done when PRO assessments are missed, including contingency plans for following up patients who miss PRO assessments and who is responsible for implementing then.<br>16. Specify whether the PRO questionnaire will be used in other languages – if so, which?<br>17. State how completed PRO questionnaires are submitted by the patient<br>**Timing of assessments:**<br>18. Specify the timeframe of interest and rationale for this. (This should be based on the expected clinical trajectory with treatment, e.g. do you expect the PRO to worsen while the patient is on treatment, then return to normal after treatment completion?)<br>19. Specify the time points at which PRO questionnaires will be administered.<br>20. Specify acceptable time windows for each assessment.<br>21. Specify which measures will be used at each assessment.<br>22. Include PRO questionnaire time points in main protocol schedule of assessments table.<br>23. Specify any stopping rule for PRO assessment (e.g. if the patient stops treatment early, how long will PRO assessment continue? Will PRO be assessed on trial exit?)			
**DATA MANAGEMENT**<br>24. Specify where will PRO questionnaire data will be stored<br>25. Specify security measures in place to ensure confidentiality of patient data<br>26. Specify what will happen to PRO data if a patient decides to exit the project			
**ENDPOINTS**<br>27. Describe methods for deriving PRO endpoints from PRO data, and which PRO assessment time-points are involved.<br>28. Describe the analysis metric, e.g. change from baseline, final value, responder (yes/no).<br>29. Where possible, reference scoring manuals for summated scales from questionnaires (domain-specific &/or total), and methodological papers for composite endpoints (e.g. QTWiST).<br>30. Describe PRO responder definitions (size and duration of benefit), where relevant.			
**STATISTICAL CONSIDERATIONS**<br>31. State the sample size considerations for PRO endpoints: EITHER<br>1) sample size calculations (if PRO is primary endpoint or if sample size for PRO as secondary endpoint differs from that for primary endpoint) OR<br>2) power calculations (if sample size for PRO as secondary endpoint same as that for primary endpoint).<br>32. State minimal important difference (with reference/s) – relevant to sample size calculations, responder definitions and interpreting clinical significance of results.<br>33. Describe the analytic plan, with reference to where other details of the statistical analysis plan can be found, if not in the protocol. These should include:			

a. State methods to calculate missing data rates and assess patterns of missing data.<br>b. Describe methods for handling missing data.<br>c. State method for adjusting statistical significance level (critical p value for Type 1 error) to allow for multiple hypothesis testing (across domains and time points)			
**REFERENCES**<br>34. Provide references for validation studies of the chosen PRO questionnaires<br>35. Provide references for what is known about PROs (as per Background and Rationale section)<br>36. Provide references for PRO data analyses and methods for handling missing data			
**APPENDICES**<br>37. PRO questionnaires.<br>38. Evidence of permission to use PRO questionnaires (where applicable. If not required, state so).<br>39. Patient Reported Outcomes (PRO) Completion and Missing Data (CoMiDa) Form – to record reasons for missing PRO data, which may inform analyses<br>40. Sample information sheet and consent form (in which the patient is informed about the requirement and purpose of PRO questionnaires in this research, who has access to the PRO data and who to contact with questions).			
