# Supplementary material for: Improving the patient-reported outcome sections of clinical trial protocols: a mixed methods evaluation of educational workshops
Source: Qual Life Res. 2022 May 12;31(10):2901–16. doi: 10.1007/s11136-022-03127-w (PMC9470723; doi:10.1007/s11136-022-03127-w)
Supplement: Supplementary file 3 — Supplementary file3 (PDF 226 kb) [file 11136_2022_3127_MOESM3_ESM.pdf]

Improving the patient-reported outcome sections of clinical trial protocols: a mixed methods evaluation of educational workshops

Madeleine T King[1*], Margaret-Ann Tait[1], Rachel Campbell[1], Fabiola Müller[1,2], Claudia Rutherford[1,3], Corinna Beckmore[4], Sophie Chima[5], Danette Langbecker[6], Joanne Shaw[7], Rebecca Mercieca-Bebber[8]

1. University of Sydney, School of Psychology, Sydney Quality of Life Office, Australia
2. Department of Medical Psychology, Amsterdam University Medical Centers, University of Amsterdam, Amsterdam Public Health Research Institute, Amsterdam, the Netherlands
3. University of Sydney, Sydney Nursing School, Cancer Nursing Research Unit (CNRU), Sydney, Australia
4. Breast Cancer Trials, Newcastle, Australia
5. Department of General Practice, Faculty of Medicine, Dentistry and Health Sciences, Centre for Cancer Research, University of Melbourne, Victorian Comprehensive Cancer Centre, Melbourne, Vic., Australia.
6. Centre for Online Health, The University of Queensland, St Lucia, Australia
7. The University of Sydney, School of Psychology, Psycho-oncology Co-operative Research Group, Sydney Australia
8. University of Sydney, NHMRC Clinical Trial Centre, Sydney, Australia

*madeleine.king@sydney.edu.au

Online supplement 3: Post-workshop evaluation survey

PROtocol Checklist Workshop 2014

Evaluation

1. Did this workshop meet your expectations? Please comment.

2. What sessions or topics of this workshop were most useful?

3. What sessions or topics of this workshop do you feel could be improved? How?

4. Would you add any sessions or specific topics to this workshop?

5. How useful is the *PROtocol Checklist* resource?

6. Did you find the real protocol examples (i.e. PLUNG, HPV and OUTBACK) to be useful?

7. Would you prefer to spend time at the end of each session working on your own protocol (reflect, review, write) or discussing your and other protocols with other participants?

8. Who from your Trials Group do you feel would benefit most from attending the PROtocol Checklist Workshop? (e.g. Lead investigators, central operations staff, etc.....)

9. Overall rating of the PROtocol Checklist Workshop (1 = poor, 10=excellent)

1	2	3	4	5	6	7	8	9	10

10. How did you find the length of the workshop?
    a) The right length (2 days)
    b) Too long – (please specify your preferred length for this workshop) ___ days
    c) Too short – (please specify your preferred length for this workshop) ___ days

11. Which of the following workshops would you be interested in attending in the future?
    a) PRO measures
    b) PRO study design and analysis
    c) Interpretation of PRO measures and results, including the minimally important difference (MID)
    d) CONSORT-PRO - guidelines for preparation of PRO publications
    e) Other QOL/PRO workshop suggestions , please specify:
    ____
