# Supplementary material for: Improving the patient-reported outcome sections of clinical trial protocols: a mixed methods evaluation of educational workshops
Source: Qual Life Res. 2022 May 12;31(10):2901–16. doi: 10.1007/s11136-022-03127-w (PMC9470723; doi:10.1007/s11136-022-03127-w)
Supplement: Supplementary file 4 — Supplementary file4 (PDF 194 kb) [file 11136_2022_3127_MOESM4_ESM.pdf]

Improving the patient-reported outcome sections of clinical trial protocols: a mixed methods evaluation of educational workshops

Madeleine T King[1*], Margaret-Ann Tait[1], Rachel Campbell[1], Fabiola Müller[1,2], Claudia Rutherford[1,3], Corinna Beckmore[4], Sophie Chima[5], Danette Langbecker[6], Joanne Shaw[7], Rebecca Mercieca-Bebber[8]

1. University of Sydney, School of Psychology, Sydney Quality of Life Office, Australia
2. Department of Medical Psychology, Amsterdam University Medical Centers, University of Amsterdam, Amsterdam Public Health Research Institute, Amsterdam, the Netherlands
3. University of Sydney, Sydney Nursing School, Cancer Nursing Research Unit (CNRU), Sydney, Australia
4. Breast Cancer Trials, Newcastle, Australia
5. Department of General Practice, Faculty of Medicine, Dentistry and Health Sciences, Centre for Cancer Research, University of Melbourne, Victorian Comprehensive Cancer Centre, Melbourne, Vic., Australia.
6. Centre for Online Health, The University of Queensland, St Lucia, Australia
7. The University of Sydney, School of Psychology, Psycho-oncology Co-operative Research Group, Sydney Australia
8. University of Sydney, NHMRC Clinical Trial Centre, Sydney, Australia

*madeleine.king@sydney.edu.au

Online supplement 4: Long-term research practice survey

Review of the Quality of Life Office PROtocol Checklist Workshop

The Quality of Life Office is supported by Cancer Australia to support the cancer clinical trials groups (CCTGs) to include patient reported outcomes in the research studies.

As part of this service, we have been running an annual PROtocol Checklist Workshop since 2011 to assist CCTG members to develop the quality of life and patient-reported outcome aspects of their trial protocols and to develop expertise among these members. Across all 7 workshops, 107 participants have attended.

We are administering this survey to obtain feedback on the long-term impact of our workshop. Please complete the short survey below – it is completely anonymous. It should take 5 minutes and will help us to improve our services in the future. Some of the questions relate to the workshop, while others relate to the PROtocol Checklist.

The checklist is available here, or can be accessed via the QOL Resources web-page: http://www.pocog.org.au/content.aspx?page=qolresources.

Do you recall attending the Quality of Life Office Protocol Checklist Workshop?

◯ Yes
◯ No

What year did you attend the PROtocol Checklist Workshop? (this was on your email invitation)

◯ 2011
◯ 2012
◯ 2013
◯ 2014
◯ 2015
◯ 2016
◯ 2017

Since the workshop, have you used the Quality of Life PROtocol Checklist:

	yes	no
In developing new protocols ?	◯	◯
In amending existing protocols?	◯	◯
In other aspects of PRO/QOL studies? e.g. implementation, analysis, interpretation, reporting, etc	◯	◯

How did you use the checklist?

____

What were the barriers to using the checklist?

____

How useful do you think this checklist would be if you were to develop a new study with patient-reported outcomes?

Not at all | Very useful

(Place a mark on the scale above)

Other comments on the PROtocol Checklist Workshop and/or the PROtocol Checklist

____
