# Supplementary material for: Improving the patient-reported outcome sections of clinical trial protocols: a mixed methods evaluation of educational workshops
Source: Qual Life Res. 2022 May 12;31(10):2901–16. doi: 10.1007/s11136-022-03127-w (PMC9470723; doi:10.1007/s11136-022-03127-w)
Supplement: Supplementary file 5 — Supplementary file5 (PDF 278 kb) [file 11136_2022_3127_MOESM5_ESM.pdf]

Improving the patient-reported outcome sections of clinical trial protocols: a mixed methods evaluation of educational workshops

Madeleine T King[1*], Margaret-Ann Tait[1], Rachel Campbell[1], Fabiola Müller[1,2], Claudia Rutherford[1,3], Corinna Beckmore[4], Sophie Chima[5], Danette Langbecker[6], Joanne Shaw[7], Rebecca Mercieca-Bebber[8]

1. University of Sydney, School of Psychology, Sydney Quality of Life Office, Australia
2. Department of Medical Psychology, Amsterdam University Medical Centers, University of Amsterdam, Amsterdam Public Health Research Institute, Amsterdam, the Netherlands
3. University of Sydney, Sydney Nursing School, Cancer Nursing Research Unit (CNRU), Sydney, Australia
4. Breast Cancer Trials, Newcastle, Australia
5. Department of General Practice, Faculty of Medicine, Dentistry and Health Sciences, Centre for Cancer Research, University of Melbourne, Victorian Comprehensive Cancer Centre, Melbourne, Vic., Australia.
6. Centre for Online Health, The University of Queensland, St Lucia, Australia
7. The University of Sydney, School of Psychology, Psycho-oncology Co-operative Research Group, Sydney Australia
8. University of Sydney, NHMRC Clinical Trial Centre, Sydney, Australia

*madeleine.king@sydney.edu.au

Online supplement 5: SPIRIT-PRO Extension Checklist

THE UNIVERSITY OF SYDNEY

SQOLO Sydney Quality Of Life Office

SPIRIT-PRO Extension checklist: recommended items to address in a clinical trial protocol regarding patient-reported outcomes (PROs)*

Trial/CCTG/PI:
General comments:

e.g. if all QOL/PRO components are in one section, give section number and title.

Protocol section	SPIRIT-PRO item		Protocol section number + specific comments
**Administrative information**			
Roles and responsibilities	SPIRIT-5a-PRO Elaboration	Specify the individual(s) responsible for the PRO content of the trial protocol.	
**Introduction**			
Background and rationale	SPIRIT-6a-PRO Extension	Describe the PRO specific research question and rationale for PRO assessment, and summarize PRO findings in relevant studies.	
Objectives	SPIRIT-7-PRO Extension	State specific PRO objectives or hypotheses (including relevant PRO concepts/domains).	
**Methods: Participants, interventions, and outcomes**			
Eligibility criteria	SPIRIT-10-PRO Extension	Specify any PRO-specific eligibility criteria (e.g., language/reading requirements or pre-randomization completion of PRO). If PROs will not be collected in the entire study sample, provide a rationale and describe the method for obtaining the PRO subsample.	
Outcomes	SPIRIT-12-PRO Extension	Specify the PRO concepts/domains used to evaluate the intervention (e.g., overall HRQOL, specific domain, specific symptom) and, for each one, the analysis metric (e.g., change from baseline, final value, time to event) and the principal time point or period of interest.	
Participant timeline	SPIRIT-13-PRO Extension	Include a schedule of PRO assessments, providing a rationale for the time points, and justifying if the initial assessment is not pre-randomization. Specify: time windows; whether PRO collection is prior to clinical assessments; and if using multiple questionnaires, whether order of administration will be standardized.	
Sample size	SPIRIT-14-PRO Elaboration	Where a PRO is the primary endpoint, state the required sample size (and how it was determined) and recruitment target (accounting for expected loss to follow-up). If sample size is not established based on PRO endpoint, then discuss the power of the principal PRO analyses.	

*Calvert M, Kyte D, Mercieca-Bebber R, Slade A, Chan AW, King MT, et al. Guidelines for Inclusion of Patient-Reported Outcomes in Clinical Trial Protocols: The SPIRIT-PRO Extension. *JAMA*. 2018;319(5):483-94.

Protocol section	SPIRIT-PRO item		Protocol section number + specific comments
**Methods: Data collection, management, and analysis**			
Data collection methods	SPIRIT-18a(i)-PRO Extension	Justify the PRO instrument to be used, and describe domains, number of items, recall period, instrument scaling/scoring (e.g., range and direction of scores indicating a good/poor outcome). Evidence of PRO instrument measurement properties, interpretation guidelines, and patient acceptability/burden should be provided or cited if available, ideally in the population of interest. State whether the measure will be used in accordance with any user manual and specify and justify deviations if planned.	
	SPIRIT-18a(ii)-PRO Extension	Include a data collection plan outlining the permitted mode(s) of administration (e.g., paper, telephone, electronic, other) and setting (e.g., clinic, home, other).	
	SPIRIT-18a(iii)-PRO Extension	Specify whether more than one language version will be used, and state whether translated versions have been developed using currently recommended methods.	
	SPIRIT-18a(iv)-PRO Extension	Where the trial context requires someone other than the trial participant to answer on their behalf (a proxy reported outcome), state and justify this. Provide/cite evidence of the validity of proxy assessment if available.	
	SPIRIT-18b(i)-PRO Extension	Specify PRO data collection and management strategies for minimising avoidable missing data.	
	SPIRIT-18b(ii)-PRO Elaboration	Describe the process of PRO assessment for participants who discontinue or deviate from their assigned intervention protocol	
Statistical methods	SPIRIT-20a-PRO Elaboration	State PRO analysis methods including any plans for addressing multiplicity/type 1 (α) error.	
	SPIRIT-20c-PRO Elaboration	State how missing data will be described and outline the methods for handling missing items or entire assessments (e.g., approach to imputation and sensitivity analyses).	
**Methods: Monitoring**			
Harms	SPIRIT-22-PRO Extension	State whether or not PRO data will be monitored during the study to inform the clinical care of individual trial participants and, if so, how this will be managed in a standardized way. Describe how this process will be explained to participants, e.g., in the participant information sheet and consent form.	
**Appendices**			
Questionnaires	N/A		

This document prepared by the Sydney Quality of Life Office was adapted with permission from 'Guidelines for Inclusion of Patient-Reported Outcomes in Clinical Trial Protocols: The SPIRIT-PRO Extension' (Calvert, et al., 2018) for use by the Collaborative Cancer Clinical Trials Groups supported by Cancer Australia.
