# Supplementary material for: Improving the patient-reported outcome sections of clinical trial protocols: a mixed methods evaluation of educational workshops
Source: Qual Life Res. 2022 May 12;31(10):2901–16. doi: 10.1007/s11136-022-03127-w (PMC9470723; doi:10.1007/s11136-022-03127-w)
Supplement: Supplementary file 6 — Supplementary file6 (PDF 286 kb) [file 11136_2022_3127_MOESM6_ESM.pdf]

Improving the patient-reported outcome sections of clinical trial protocols: a mixed methods evaluation of educational workshops

Madeleine T King[1*], Margaret-Ann Tait[1], Rachel Campbell[1], Fabiola Müller[1,2], Claudia Rutherford[1,3], Corinna Beckmore[4], Sophie Chima[5], Danette Langbecker[6], Joanne Shaw[7], Rebecca Mercieca-Bebber[8]

1. University of Sydney, School of Psychology, Sydney Quality of Life Office, Australia
2. Department of Medical Psychology, Amsterdam University Medical Centers, University of Amsterdam, Amsterdam Public Health Research Institute, Amsterdam, the Netherlands
3. University of Sydney, Sydney Nursing School, Cancer Nursing Research Unit (CNRU), Sydney, Australia
4. Breast Cancer Trials, Newcastle, Australia
5. Department of General Practice, Faculty of Medicine, Dentistry and Health Sciences, Centre for Cancer Research, University of Melbourne, Victorian Comprehensive Cancer Centre, Melbourne, Vic., Australia.
6. Centre for Online Health, The University of Queensland, St Lucia, Australia
7. The University of Sydney, School of Psychology, Psycho-oncology Co-operative Research Group, Sydney Australia
8. University of Sydney, NHMRC Clinical Trial Centre, Sydney, Australia

*madeleine.king@sydney.edu.au

Online supplement 6: Guide for review of protocols against the PROtocol and SPIRIT-PRO Checklists

PROtocol Checklist Workshop Evaluation - Protocol Evaluation Rating Anchors

Please use the following verbal anchors to guide your rating.

- N/A marks items that can be 'not applicable' in a certain protocol

If you experience any difficulties using these anchors for a specific item in a specific protocol, please explain why in the comments column in the PCW Evaluation Checklist spreadsheet.

**SPIRIT-PRO Checklist Item**	**PROtocol Checklist Item**	**Scoring**		
		**0**	**1 - 9**	**10**
	**COLLABORATORS / SUMMARY / BACKGROUND / OBJECTIVES**			
**5a**	**1**	Individual not named anywhere in the protocol.	[answer 0 *or* 10 only]	Individual is named in the protocol (reference to QOL/PRO or protocol specific term, e.g. pain).
**-**	**2**	*None* of the 4 subitems are addressed.	Rate the extent to which *all* of the 4 subitems are clearly addressed.	*All* 4 subitems are clearly addressed.
**-**	**3**	No mention of PRO assessment in study schema *or* assessment schedule in the protocol summary/synopsis.	[answer 0 *or* 10 only]	PRO assessment mentioned in study schema *or* assessment schedule in the protocol summary/synopsis.
**6a_ii**	**5**	No literature relevant to PROs is cited.	Use your discretion to rate how comprehensive the literature review is with regard to the relevant clinical context.	A comprehensive literature review about PROs in this clinical context is provided.
**6a_i_y**	**6**	No explicit rationale for PRO assessment is provided.	Rate the extent to which a clear and comprehensive rationale for PRO assessment is explicitly provided .	A clear and comprehensive rationale for PRO assessment is explicitly provided.
**6a_i_z**	**-**	No explicit PRO-specific research question is provided.	Rate the extent to which a clearly defined PRO-specific research explicitly provided.	A clearly defined and precise PRO-specific research question is provided.
**7**	**4**	No explicit PRO-specific objective(s) or hypothesis/es stated.	Rate the extent to which PRO-objective(s) or hypothesis/es are clearly defined with reference to *all key* PRO-domains.	Clearly defined PRO-objective(s) or hypothesis/es stated with reference to *all key* PRO-domains.
**-**	**4**	No time-points stated in PRO-specific objective(s) *or* hypothesis/es.	Use your discretion to rate how completely and specifically the time points are stated (e.g. referring to weeks/months, beginning/end treatment, etc.).	Time points clearly stated in PRO-specific objective(s) *and* hypothesis/es.

	**METHODS**					
**10_i**	**8**	No mention of the issue of eligibility criteria for PRO endpoints/substudy.		[answer 0 *or* 10 only]		The eligibility of trial participants is stated (e.g. “all participants are eligible for PRO assessment” or PRO-specific eligibility criteria are provided).
**10_ii**	**-**	*Neither* rationale *nor* method for collecting the PRO-subsample is stated.<br><or **N/A**>		Rate the extent to which *both* the rationale and the method are provided and how clearly they are stated.<br><or **N/A**>		Rationale and method for collecting the PRO-subsample are *both* clearly stated.
**12_i**	**7**	PRO concepts/domains used to evaluate the intervention are not stated.		Rate the extent to which *all* relevant concepts and domains are specifically stated (e.g. “quality of life” is too vague to rate highly, specific domains of interest must be stated, e.g. “fatigue, pain, social functioning”); ideally, these should align with the trial objectives.		*All* PRO concepts/domains used to evaluate the intervention are specifically stated; ideally, these should align with the trial objectives.
	**QUESTIONNAIRES**					
**18ai_z**	**9**	No justification provided for *each* PRO instrument selected.		Rate the extent to which the justification addresses *all* PRO instruments selected and their suitability for the clinical context.		Clear justification provided for why *each* PRO instrument has been selected for this clinical context.
**18ai_y**	**10**	*None* of the 4 subitems are addressed for *each* PRO instrument.		Rate the extent to which *all* 4 subitems are clearly and fully addressed for *each* PRO instrument.		*All* 4 subitems are clearly addressed for *each* PRO instrument.
**18ai_x**	**11**	*None* of the 3 subitems are addressed for *each* PRO instrument. Note that interpretation guidelines and information about patient burden and acceptability might not be available.		Rate the extent to which *at least* the measurement properties are addressed for *all* PRO instrument(s) in this clinical context. Note that interpretation guidelines and information about patient burden and acceptability might not be available.		*All* 3 subitems are clearly addressed for *each* PRO instrument in this clinical context. Since interpretation guidelines and information about patient burden and acceptability might not be available for *all* PRO instruments a statement to this effect is sufficient to score 10.
**18ai_w**	**-**	No mention of whether the PRO(s) will be used in accordance with the user manual and *no* justification for *any* deviation(s) is provided.		Rate whether it is clearly stated that *each* PRO instrument will be used in accordance with the user manual and how clearly *any* deviation(s) is justified.		States clearly whether *all* PRO instruments will be used in accordance with the user manual. *Any* deviation(s) is clearly and fully justified.
	**ADMINISTRATION**					

**18aii_z**	**12**	The mode(s) of PRO administration permitted in this trial is not stated.		Rate the extent to which the mode(s) of administration is explicitly stated (e.g. “participants should be able to write” implies hard-copy but is too vague to rate highly; “all questionnaires will be administered in paper-and-pencil form” is specific).		The mode(s) of PRO administration permitted in this trial is explicitly stated.
**-**	**13**	The person responsible for administering and retrieving the PRO questionnaire(s) or for sending reminders is not specified.		Rate how clearly the protocol specifies who the person responsible is (e.g. “site/hospital staff” or “project member” is too vague to rate highly; “trained nurse” is specific).		The person responsible for the administering and retrieving the PRO questionnaire(s) or for sending reminders is clearly specified.
**18aii_y**	**14**	The setting of PRO data collection is not specified.		[answer 0 *or* 10 only]		The setting of PRO data collection is specified (e.g. clinic, home, etc.).
**18bEx**	**-**	Strategies for minimising avoidable[1] missing data are not specified.		Rate the extent to which strategies are specified for addressing common reasons for avoidable missing data (e.g. administrative errors, lack of explanation of importance of PRO data, burdensome questionnaires).		Strategies for minimising avoidable missing data are clearly specified.
**-**	**15**	Does not specify what should be done if PRO assessments are missed.		Rate the extent to which it is clearly specified what should be done if PRO assessments are missed and who is responsible for implementing this plan (e.g. plans for following patients who miss PRO assessments).		Clearly specifies what should be done if PRO assessments are missed and who is responsible for implementing this plan.
**18aiii_z**	**16**	Does not specify whether more than one language version will be used.		Rate how explicitly language version(s) is specified (e.g. stating that English is an eligibility criteria is too vague to rate highly; stating that questionnaires will *only* be provided in English is explicit).		Explicitly specifies whether more than one language version will be used, and which languages will be used.
**18aiii_y**	**-**	A language translation(s) will be used but the method for translation is not stated.<br><or **N/A**>		[answer 0 *or* 10 only]<br><or **N/A**>		States that the language translation(s) to be used was developed using currently recommended method(s) (e.g. “own translation” is not a recommended method; “validated translations” are recommended).

[1]Not all missing PRO data are avoidable: patients have the right to decide not to complete questionnaires. Common reasons for avoidable missing PRO data are administrative errors, lack of explanation of the importance of PRO data, and overly burdensome questionnaires.

**18aiv**	**-**	A proxy reported outcome is to be used, but its use is not justified and no evidence for its validity is provided.<br><or **N/A**>		Rate whether the use of the proxy reported outcome is clearly justified and whether sufficient evidence for its validity is provided (e.g. cites a study demonstrating its validity).<br><or **N/A**>		A proxy reported outcome is to be used, its use is clearly justified, and evidence for its validity is provided.
	**TIMING of ASSESSMENTS**					
**13_i**	**19, 21, 22**	A schedule for PRO assessment(s) is not provided.		A schedule for PRO assessment(s) is provided but it does not cover *all* PRO measure(s) and time point(s). Rate how comprehensively the schedule covers *all* measure(s) *and* time point(s).		The schedule for PRO assessment(s) covers *all* PRO measure(s) *and* time point(s).
**13_ii**	**18**	A rationale for the PRO assessment time points is not provided.		Rate the extent to which a clear rationale is provided for *each* assessment time point (e.g. there are 3 time points but only 1 justified, is not sufficiently comprehensive to rate highly; if 2 out of 3 are justified, it would rate more highly).		A clear rationale for *all* PRO assessment time points is provided (e.g. there are 3 time points and each is justified as follows: "pre-randomization baseline will avoid psychological bias", "end of treatment is when maximum toxicity is expected", "6 weeks after treatment is when maximum palliative benefit is expected"; would rate a 10).
**13_iii**	**-**	The initial PRO assessment is post-randomization and no justification is provided.<br><or **N/A**>		[answer 0 *or* 10 only]<br><or **N/A**>		The initial PRO assessment is pre-randomization *or* the initial PRO assessment is post-randomization and a justification is provided.
**13_iv**	**20**	The time window(s) are not specified for *any* PRO assessment(s) time points.		Rate to what extent a specific time window is specified for *all* of the PRO assessment(s) (e.g. there are 3 time points but only 1 time window specified, is not sufficiently comprehensive to rate highly; if 2 out of 3 time points have specified time windows it would rate more highly).		A specific time window is specified for *all* PRO assessment(s) time points (e.g. there are 3 time points and a time window is clearly specified for *each*: baseline assessment is "pre-surgery", the time window is from "1 week to 1 day prior to surgery"; for "post-surgery" assessment, the time window is "day of discharge to 1 week post-discharge"; for "1-year follow-up" the time window is "10 to 14 months after date of surgery").

**13_v**	**-**	It is not specified whether the PRO collection is *prior* to clinical assessment(s).<br><or **N/A**>		[answer 0 *or* 10 only]<br><or **N/A**>		It is explicitly specified whether the PRO collection is *prior* to clinical assessment(s).
**13_vi**	**-**	Multiple questionnaires are used but it is not specified whether the order of PRO administration will be standardized.<br><or **N/A**>		[answer 0 *or* 10 only]<br><or **N/A**>		Multiple questionnaires are used, and their order of administration is specified, i.e. standardized (e.g. there are 2 questionnaires (A and B) and it is clearly specified that A must be completed before B; randomised order is also a valid standardisation, easily implemented with electric mode of data collection).
**18bEl**	**23**	The process of PRO assessment for participants who discontinue/deviate is not described.		Rate to what extent the process of PRO assessment for participants who discontinue/deviate is explicitly described (e.g. “no further information will be collected” is too vague to rate highly).		The process of PRO assessment for participants who discontinue/deviate is explicitly described (e.g. “participants who discontinue the intervention must complete a study exit assessment; they will be contacted twice to enquire about willingness to fill in the follow-up questionnaire”).
	**DATA MANAGEMENT**					
**-**	**24**	It is not specified where PRO data will be stored.		[answer 0 *or* 10 only]		It is explicitly specified where PRO data will be stored.
**-**	**25**	Security measures to ensure confidentiality of patient data are not specified.		Rate the extent to which security measure(s) to ensure confidentiality are specifically described (e.g. storage of data in a secure access-restricted area, reducing the identifiability of data, application of data encryption).		A comprehensive range of security measures is specified to ensure confidentiality of patient data, such as storage of data in a secure access-restricted area, reducing the identifability of data, application of data encryption.
**-**	**26**	The protocol does not specify what will happen to PRO data if patients exit the study.		[answer 0 *or* 10 only]		The protocol specifies what will happen to PRO data if patients exit the study.
**22_i**	**-**	It is not stated whether PRO data will be monitored to inform clinical care.		[answer 0 *or* 10 only]		It is stated whether PRO data will be monitored to inform clinical care.
**22_i_z**	**-**	PRO data will be monitored to inform clinical care, but it is not stated how it will be managed.<br><or **N/A**>		[answer 0 *or* 10 only]<br><or **N/A**>		PRO data will be monitored to inform clinical care, and it is stated how it will be managed in a standardized way.

**22_ii**	**-**	PRO data will be monitored to inform clinical care, but the protocol does not describe how this process will be explained to participants.<br><or **N/A**>		[answer 0 *or* 10 only]<br><or **N/A**>		PRO data will be monitored to inform clinical care, and the protocol describes how this process will be explained to participants.
	**ENDPOINTS**					
**-**	**27(a)**	The method(s) for deriving PRO endpoint(s) from PRO data is not described.		Rate to what extent the method(s) for deriving PRO endpoints (i.e. primary and/or secondary) are clearly described for *all* PROs.		The method(s) for deriving *all* PRO endpoint(s) from PRO data is clearly described. For *each* PRO endpoint, this would include the specific PRO domain (e.g. pain), the time points (e.g. baseline, end of palliative radiotherapy) and the method (e.g. calculate change score from baseline to end of treatment, *or* calculate the proportion of patients improved by a clinically important degree at end of treatment).
**12_ii_z**	**28**	The analysis metric used to evaluate the intervention is not specified for *any* PRO(s).		Rate to what extent the analysis metric is specified for *all* PRO(s).		The analysis metric used to evaluate the intervention is clearly specified for *all* PRO(s) (e.g. change score from baseline to end of treatment, *or* the proportion of patients clinically improved at end of treatment).
**12_ii_y**	**27 (b)**	The time point used to evaluate the intervention is not specified for *any* PRO(s).		Rate how specifically the time point is stated for *all* PRO(s) (e.g. “follow-up” is too vague to rate highly; “2 weeks after treatment completion” would rate highly).		The time point used to evaluate the intervention is clearly specified for *all* PRO(s).
**- similar to Spirit-PRO-12**	**29**	The scoring manuals for summated scales (and methodological papers for composite endpoints where applicable) are not referenced. Note that the scoring manuals might not be available for some PROs.		[answer 0 *or* 10 only]		The scoring manuals for summated scales (and methodological papers for composite endpoints where applicable) are referenced. Note that the scoring manuals might not be available for some PROs. A statement to this effect is sufficient to score 10.
**- similar to Spirit-PRO-12**	**30**	A PRO responder definition(s) is used in *at least* one endpoint, but a responder definition is not provided.<br><or **N/A**>		Rate how clearly the responder definition(s) is specified for *each* PRO in terms of size and duration of the benefit.<br><or **N/A**>		The PRO responder definition(s) is clearly defined for *all* relevant PROs, both in terms of size and duration of benefit (e.g. in a trial of palliative radiotherapy, an improvement of at least 2 points on a 0-10 pain scale 6 weeks after

						end of radiotherapy will be considered a "responder").
	**STATISTICAL CONSIDERATIONS**					
**14**	**31**	PRO is a *primary* outcome, but the required sample size and the recruitment target are not specified.<br><br>*Or*<br><br>PRO is a *secondary* outcome, but the power for the principle PRO is not discussed.		If PRO is a *primary* outcome, rate how clearly the following points are addressed:<br>- The required sample size<br>- How the required sample size was determined<br>- The recruitment target accounting for expected loss to follow-up<br><br>If PRO is a *secondary* outcome, rate how clearly the power for the principle PRO is discussed.		PRO is a *primary* outcome and the required sample size, how it was determined, the target sample size and the expected loss to follow-up are clearly specified.<br><br>*Or*<br><br>PRO is a *secondary* outcome and the power for the principle PRO is clearly discussed.
**-**	**32**	The minimal important difference is not stated for *any* PRO(s).		[answer 0 *or* 10 only]		The minimal important difference is stated for *at least the key* PRO(s) (i.e. those stated as likely to be affected by the intervention).
**20a_i**	**33**	There is no mention of PRO analysis method(s).		Rate the extent to which the analysis method(s) is comprehensively described. A comprehensive description covering statistical technique (e.g. t-test, ANOVA, model type) and other considerations (e.g. covariates) would rate highly.		The analysis method(s) for *at least* the *key* PRO(s) is clearly and comprehensively stated.
**20c_i**	**33a**	It is not stated how missing data (i.e. missing items and entire assessments) will be described.		[answer 0 *or* 10 only]		It is stated how missing data (i.e. missing items and entire assessments) will be described.
**20c_ii**	**33b**	There is no mention of how missing data (i.e. missing items and entire assessments) will be handled.		Rate the extent to which the methods for handling *both* missing items and entire assessments are stated.		The methods for handling *both* missing items and entire assessments are stated.
**20a_ii**	**33c**	No mention of plan(s) for addressing multiplicity/type 1 (α) error is stated.		[answer 0 *or* 10 only]		The plan(s) for addressing multiplicity/type 1 (α) error is stated.
	**REFERENCES / APPENDICES**					
**n/a**	**35**	No PRO-specific references are provided.		[answer 0 *or* 10 only]		PRO-specific references are provided.

**n/a**	**36**	No references for PRO data analysis and methods for handling missing data are provided.		[answer 0 *or* 10 only]		References for PRO data analysis and methods for handling missing data are provided.
**n/a**	**37**	Copy/ies of PRO questionnaire(s) are not provided.		[answer 0 *or* 10 only]		Copy/ies of PRO questionnaire(s) are provided.
**n/a**	**38**	Evidence of permission to use PRO questionnaires is not provided. Note that permission might not be required for some PROs.		[answer 0 *or* 10 only]		Evidence of permission to use PRO questionnaires is provided. Note that permission might not be required for some PROs. A statement to this effect is sufficient to score 10.
**n/a**	**39**	A copy of the CoMiDa form is not provided.		[answer 0 *or* 10 only]		A copy of the CoMiDa form is provided.
**n/a**	**40**	The requirement and purpose of PRO questionnaires is not mentioned in the Patient Information Sheet (PIS) or Consent Form (CF). Also score 0 if the PIS/CF are not provided.		[answer 0 *or* 10 only]		The requirement and purpose of PRO questionnaires is mentioned in the Patient Information Sheet (PIS) or Consent Form (CF).
