# Supplementary material for: Improving the patient-reported outcome sections of clinical trial protocols: a mixed methods evaluation of educational workshops
Source: Qual Life Res. 2022 May 12;31(10):2901–16. doi: 10.1007/s11136-022-03127-w (PMC9470723; doi:10.1007/s11136-022-03127-w)
Supplement: Supplementary file 7 — Supplementary file7 (PDF 242 kb) [file 11136_2022_3127_MOESM7_ESM.pdf]

Improving the patient-reported outcome sections of clinical trial protocols: a mixed methods evaluation of educational workshops

Madeleine T King[1*], Margaret-Ann Tait[1], Rachel Campbell[1], Fabiola Müller[1,2], Claudia Rutherford[1,3], Corinna Beckmore[4], Sophie Chima[5], Danette Langbecker[6], Joanne Shaw[7], Rebecca Mercieca-Bebber[8]

1. University of Sydney, School of Psychology, Sydney Quality of Life Office, Australia
2. Department of Medical Psychology, Amsterdam University Medical Centers, University of Amsterdam, Amsterdam Public Health Research Institute, Amsterdam, the Netherlands
3. University of Sydney, Sydney Nursing School, Cancer Nursing Research Unit (CNRU), Sydney, Australia
4. Breast Cancer Trials, Newcastle, Australia
5. Department of General Practice, Faculty of Medicine, Dentistry and Health Sciences, Centre for Cancer Research, University of Melbourne, Victorian Comprehensive Cancer Centre, Melbourne, Vic., Australia.
6. Centre for Online Health, The University of Queensland, St Lucia, Australia
7. The University of Sydney, School of Psychology, Psycho-oncology Co-operative Research Group, Sydney Australia
8. University of Sydney, NHMRC Clinical Trial Centre, Sydney, Australia

*madeleine.king@sydney.edu.au

Online supplement 7: Inter-rater reliability - Weighted Kappa (linear weights)

Sub-item no.	Descriptive label	Weighted Kappa	P value	95% CI
1	Specify the individual(s) responsible for the PRO content of the trial protocol.	.000 [a]	/	/
2	In the protocol summary - Identify specific PRO endpoint(s), specifying key PRO construct(s)/domain(s), time-point(s), analysis metric(s). (i.e. change in score)	0.524	<.001*	0.294;0.755
3	In the protocol summary - PRO assessment included in the study schema / assessment schedule.	0.182	0.375	-0.214;0.577
4	Summarize PRO findings of past relevant studies.	0.349	.025*	0.056;0.642
5	Describe the rationale for PRO assessment.	0.471	.002*	0.218;0.724
6	Describe the PRO specific research question.	0.466	.002*	0.224;0.708
7	State specific PRO objectives or hypotheses (including relevant PRO concepts/domains).	0.129	0.326	-0.167;0.425
8	Do the stated PRO objectives/hypotheses include time-points?	0.45	.003*	0.138;0.762
9	Specify any PRO-specific eligibility criteria (e.g., language/reading requirements or pre-randomization completion of PRO).	0.494	.012*	0.165;0.823
10	If PROs will not be collected in the entire study sample, provide a rationale and describe the method for obtaining the PRO subsample. (If PROs are collected in the entire sample, then rate as 'N/A')	0.516	.001*	0.088;0.943
11	Specify the PRO concepts/domains used to evaluate the intervention (e.g., overall HRQOL, specific domain, specific symptom).	0.358	.007*	0.129;0.587
12	Justify the PRO instrument to be used.	0.316	.007*	0.053;0.579
13	Describe the PRO instrument in terms of domains, number of items, recall period, instrument scaling/scoring (eg, range and direction of scores indicating a good/poor outcome).	0.401	.001*	0.115;0.686
14	Evidence of PRO instrument measurement properties, interpretation guidelines, and patient acceptability/burden should be provided or cited if available, ideally in the population of interest.	0.41	.003*	0.161;0.660
15	State whether the measure will be used in accordance with any user manual and specify and justify deviations if planned.	0.523	.003*	0.156;0.889
16	Include a data collection plan outlining the permitted mode(s) of administration (e.g., paper, telephone, electronic, other).	0.674	<.001*	0.458;0.890
17	Specify who is responsible for delivering PRO questionnaires to patients and retrieving completed questionnaires from them, or if online, who is responsible for sending reminders.	0.464	.002*	0.220;0.708
18	Specify PRO data collection setting (e.g., clinic, home, other).	0.607	.004*	0.261;0.953
19	Specify PRO data collection and management strategies for minimising avoidable missing data.	0.429	.003*	0.185;0.673
20	Specify what should be done when PRO assessments are missed, including contingency plans for following up patients who miss PRO assessments and who is responsible for implementing them.	0.537	<.001*	0.356;0.718
21	Specify whether more than one language version will be used.	0.397	.018*	0.113;0.680

Sub-item no.	Descriptive label	Weighted Kappa	P value	95% CI
22	If a translation will be used, state whether it was developed using currently recommended methods. (If only one language version will be used, then rate as 'N/A')	0.366	.026*	-0.076;0.808
23	Where the trial context requires someone other than the trial participant to answer on their behalf (a proxy reported outcome), state and justify this. Provide/cite evidence of the validity of proxy assessment if available. (If there are no proxy assessments, then rate as 'N/A')	0.697	<.001*	0.420;0.973
24	Include a schedule of PRO assessments, specifying which measures will be used at each assessment.	0.126	0.461	-0.383;0.635
25	Provide a rationale for the assessment time points.	0.139	0.345	-0.173;0.450
26	It is good practice to do the initial PRO assessment prior to randomization. If initial PRO assessment occurs post-randomization, provide a justification. It is good practice to do the initial PRO assessment prior to randomization. If initial PRO assessment occurs post-randomization, provide a justification. (If initial assessment is pre-randomization, rate as '10'; if initial assessment is post-randomization with a justification, rate as '10'; if initial assessment is post-randomization with no justification, rate as '0')	0.612	<.001*	0.333;0.891
27	Specify PRO assessment time windows.	0.212	0.101	-0.060;0.483
28	Specify whether PRO collection is prior to clinical assessments.	0.389	.006*	0.073;0.704
29	If using multiple questionnaires, specify whether order of administration will be standardized. (If only one questionnaire will be used, then rate as 'N/A')	0.745	<.001*	0.415;1.074
30	Describe the process of PRO assessment for participants who discontinue or deviate from their assigned intervention protocol.	0.3	.028*	0.015;0.584
31	Specify where PRO questionnaire data will be stored.	0.454	.023*	0.096;0.812
32	Specify security measures in place to ensure confidentiality of patient data.	0.268	.042*	0.026;0.0510
33	Specify what will happen to a patient's PRO data if that patient decides to exit the study.	0.614	.004*	0.127;1.101
34	State whether or not PRO data will be monitored during the study to inform the clinical care of individual trial participants.	0.834	<.001*	0.511;1.150
35	If PRO data will be monitored during the study to inform clinical care of individual participants, state how this will be managed in a standardized way. (If not monitored to inform clinical care, then rate as 'N/A')	0.731	<.001*	0.433;1.030
36	If PRO data will be monitored during the study to inform clinical care of individual participants, describe how this process will be explained to participants, e.g., in the participant information sheet and consent form. (If not monitored to inform clinical care, then rate as 'N/A')	0.642	<.001*	0.338;0.947
37	Describe methods for deriving PRO endpoints from PRO data.	0.414	.007*	0.164;0.664
38	For each of the PRO concepts/domains used to evaluate the intervention, specify the analysis metric (e.g., change from baseline, final value, time to event).	0.541	<.001*	0.326;0.756
39	For each of the PRO concepts/domains used to evaluate the intervention, specify the principal time point or period of interest.	0.412	.012*	0.127;0.697

Sub-item no.	Descriptive label	Weighted Kappa	P value	95% CI
40	Where possible, reference scoring manuals for summated scales from questionnaires (domain-specific &/or total), and methodological papers for composite endpoints (e.g. QTWiST).	-0.073	0.648	-0.186;0.040
41	Describe PRO responder definitions (size and duration of benefit), where relevant.	0.13	0.399	-0.175;0.436
42	Where a PRO is the primary endpoint, state the required sample size (and how it was determined) and recruitment target (accounting for expected loss to follow-up). If sample size is not established based on PRO endpoint, then discuss the power of the principal PRO analyses.	0.71	<.001*	0.450;0.970
43	State minimal important difference (with reference/s) – relevant to sample size calculations, responder definitions and interpreting clinical significance of results.	0.304	0.15	-0.167;0.775
44	State PRO analysis methods.	0.452	.002*	0.242;0.661
45	State how missing data will be described.	.000[a]	/	/
46	Outline the methods for handling missing items and entire assessments (e.g., approach to imputation and sensitivity analyses).	0.589	<.001*	0.251;0.926
47	State any plans for addressing multiplicity/type 1 (α) error.	0.592	.002*	0.201;0.984
48	Provide references for what is known about PROs (as per Background and Rationale section)	0.469	.022*	0.108;0.831
49	Provide references for PRO data analyses and methods for handling missing data	0.353	.030*	-0.027;0.733
50	Provide copies of PRO questionnaires.	0.897	<.001*	0.700;1.093
51	Provide evidence of permission to use PRO questionnaires (if permission not required, this is stated).	0.06	0.421	-0.059;0.178
52	Provide copies of the Patient Reported Outcomes (PRO) Completion and Missing Data (CoMiDa) Form – to record reasons for missing PRO data, which may inform analyses	1	<.001*	1.00;1.00
53	Provide sample Patient Information Sheet and Consent form (in which the patient is informed about the requirement and purpose of PRO questionnaires in this research, who has access to the PRO data and who to contact with questions).	0.577	.008*	0.152;1.002

a. Could not be calculated because R1 rating is constant (i.e. all zeros)
* indicates statistical significance
